# Albumin-Binding PSMA Radioligands: Impact of Minimal Structural Changes on the Tissue Distribution Profile

**DOI:** 10.3390/molecules25112542

**Published:** 2020-05-29

**Authors:** Luisa M. Deberle, Viviane J. Tschan, Francesca Borgna, Fan Sozzi-Guo, Peter Bernhardt, Roger Schibli, Cristina Müller

**Affiliations:** 1 Center for Radiopharmaceutical Sciences ETH-PSI-USZ, Paul Scherrer Institute, 5232 Villigen-PSI, Switzerland; luisa.deberle@psi.ch (L.M.D.); viviane.tschan@psi.ch (V.J.T.); francesca.borgna@psi.ch (F.B.); fan.sozzi@psi.ch (F.S.-G.); roger.schibli@psi.ch (R.S.); 2 Department of Radiation Physics, The Sahlgrenska Academy, University of Gothenburg, 413 45 Gothenburg, Sweden; peter.bernhardt@gu.se; 3 Department of Medical Physics and Medical Bioengineering, Sahlgrenska University Hospital, 413 45 Gothenburg, Sweden; 4 Department of Chemistry and Applied Biosciences, ETH Zurich, 8093 Zurich, Switzerland

**Keywords:** prostate cancer, PSMA ligands, lutetium-177, albumin-binder, ibuprofen, linker

## Abstract

The concept of using ibuprofen as an albumin-binding entity was recently demonstrated by the development of [^177^Lu]Lu-Ibu-PSMA-01. In the present study, we designed a novel ibuprofen-containing radioligand (Ibu-PSMA-02) with subtle structural changes regarding the linker entity in order to investigate a potential impact on the in vitro and in vivo properties. Ibu-PSMA-02 was prepared using solid-phase synthesis techniques and labeled with lutetium-177. [^177^Lu]Lu-Ibu-PSMA-02 was evaluated in vitro with regard to its plasma protein-binding properties, PSMA affinity and uptake into PSMA-expressing PC-3 PIP tumor cells. The tissue distribution profile of [^177^Lu]Lu-Ibu-PSMA-02 was assessed in tumor-bearing mice and dose estimations were performed. The in vitro characteristics of [^177^Lu]Lu-Ibu-PSMA-02 were similar to those previously obtained for [^177^Lu]Lu-Ibu-PSMA-01 with respect to plasma protein-binding, PSMA affinity and tumor cell uptake. The in vivo studies revealed, however, an unprecedentedly high uptake of [^177^Lu]Lu-Ibu-PSMA-02 in PC-3 PIP tumors, resulting in an increased absorbed tumor dose of 7.7 Gy/MBq as compared to 5.1 Gy/MBq calculated for [^177^Lu]Lu-Ibu-PSMA-01. As a consequence of the high tumor accumulation, [^177^Lu]Lu-Ibu-PSMA-02 showed higher tumor-to-background ratios than [^177^Lu]Lu-Ibu-PSMA-01. This study exemplified that smallest structural changes in the linker entity of PSMA radioligands may have a significant impact on their pharmacokinetic profiles and, thus, may be applied as a means for ligand design optimization.

## 1. Introduction

The prostate-specific membrane antigen (PSMA) was found to be overexpressed in the majority of prostate cancer cases [1,2] and is, therefore, a promising target for radionuclide therapy of metastatic castration-resistant prostate cancer (mCRPC) [3,4,5]. A wide variety of PSMA-targeting radioligands were developed in recent years, among them also conjugates for labeling with a variety of therapeutic radionuclides [6,7]. [^177^Lu]Lu-PSMA-617 is the currently best-known PSMA radioligand for therapeutic application and employed in a Phase III clinical study (VISION, NCT03511664) [8].

The concept of modifying radiopharmaceuticals with an albumin-binding entity has recently gained growing interest [9]. PSMA-targeting radioligands modified with these entities showed enhanced blood circulation in comparison to [^177^Lu]Lu-PSMA-617 and, as a consequence, considerably increased accumulation in the tumor tissue, which correlated with better therapeutic outcomes in preclinical settings [10,11,12]. The most widely used albumin binder for the modification of PSMA-targeting radioligands is the *p*-iodophenyl entity, previously discovered to bind with high affinity to serum albumin [13]. Another successfully applied albumin-binding entity is the azo dye Evans blue [14,15].

Increased blood activity levels of albumin-binding PSMA radioligands led, however, also to a higher uptake of activity in non-targeted organs and tissues including the kidneys, which may be associated with an increased risk of bone marrow and kidney toxicity. In our group, it was revealed that PSMA radioligands derivatized with entities that have lower albumin-binding affinity such as the *p*-tolyl entity [13] or ibuprofen showed more promising preclinical results [16,17]. 

Not only the nature of the albumin binder, but also the incorporation of linker entities between the PSMA ligand’s backbone and the albumin-binding group, may strongly influence the tissue distribution profile of the radioligand. It was previously observed that the hydrophilicity, length and charge of the incorporated linker play a crucial role in this regard [11,17,18]. Benešová et al. investigated the impact of negatively charged aspartate groups on a *p*-iodophenyl-containing PSMA radioligand and observed a higher kidney accumulation with an increasing number of negative charges [11]. Another study, in which PSMA-targeting radioligands were modified with hydrophilic PEG-spacers, showed a directly proportional correlation of the linker length to the clearance rate of the radioligand [18]. These examples impressively illustrated the options for fine-tuning the pharmacokinetics of PSMA radioligands.

Recently, we described PSMA radioligands modified with ibuprofen as an albumin-binding entity [17]. The albumin binder was conjugated to the radioligand’s backbone via a lysine side chain and variable amino acid-based linkers. The charge of the linker entity had a significant impact on the radioligand’s tissue distribution profile. A negatively charged linker increased the albumin-binding properties, resulting in higher background activity, whereas a positively charged amino acid led to a more efficient clearance of the radioligand from the blood [17].

**Figure 1:**
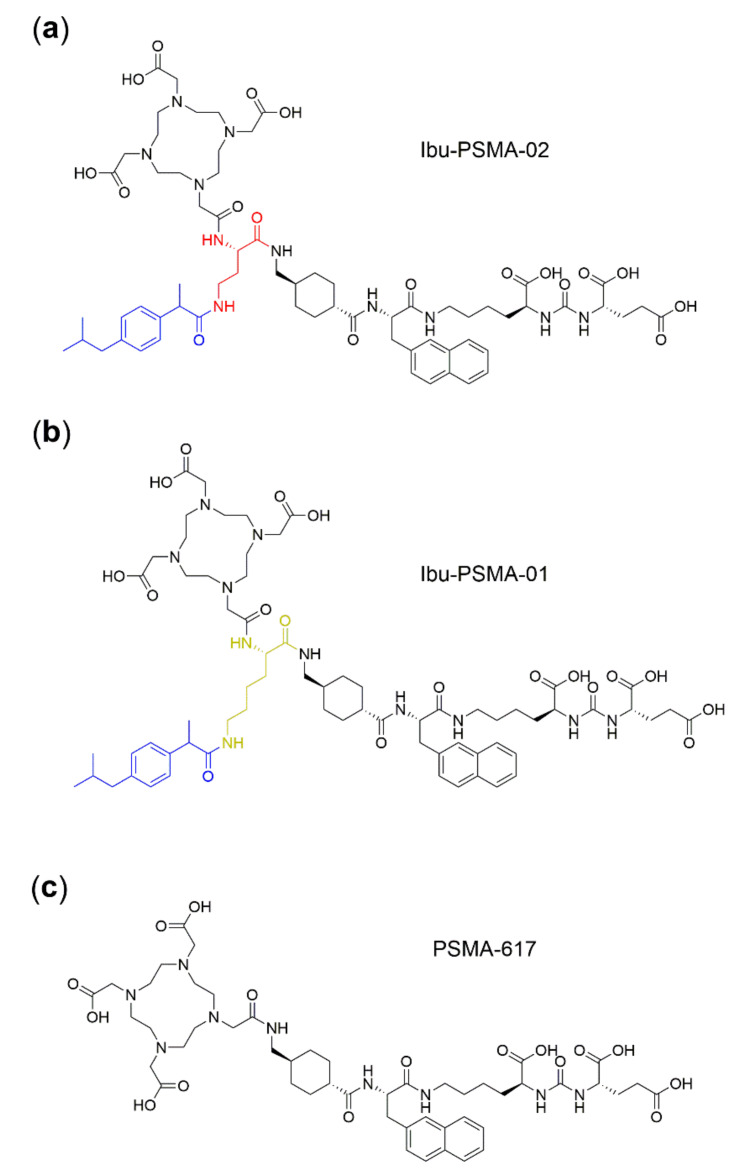
Chemical structure of prostate-specific membrane antigen (PSMA) ligands. (**a**) Novel ligand, Ibu-PSMA-02, with a diaminobutyric acid entity as connecting entity (red) to conjugate ibuprofen (blue); (**b**) Previously developed Ibu-PSMA-01 with a lysine entity as a connecting entity (yellow) to conjugate ibuprofen (blue) [17] and (**c**) PSMA-617 [11] as a reference compound without an albumin-binding entity.

The aim of the present study was to investigate whether only a subtle structural modification of the linker entity would have an impact on the radioligand’s pharmacokinetics. Instead of a lysine residue, which was the connecting entity in Ibu-PSMA (herein referred to as Ibu-PSMA-01), a diaminobutyric acid entity was used as a shorter linker for the connection of ibuprofen to the PSMA ligand’s backbone. The novel PSMA ligand, referred to as Ibu-PSMA-02, was synthesized and radiolabeled with lutetium-177 (T_1/2_ = 6.65 d; Eβ^¯^_av_ = 134 keV, Eγ = 113 keV, 208 keV) and preclinically evaluated. [^177^Lu]Lu-Ibu-PSMA-02 was characterized in vitro using PC-3 PIP/flu tumor cells and in vivo using tumor-bearing mice. The data of [^177^Lu]Lu-Ibu-PSMA-02 were compared with those previously obtained with [^177^Lu]Lu-Ibu-PSMA-01 and put in relation to the properties of [^177^Lu]Lu-PSMA-617 (Figure 1).

## 2. Results

### 2.1. Synthesis of Ibu-PSMA-02

Ibu-PSMA-02 was obtained with a yield of 15% after HPLC purification. Characterization by analytical HPLC and MALDI-TOF-MS confirmed the identity of the product and a chemical purity of >99% (Table 1, Supplementary Materials).

### 2.2. Radiolabeling and Radiolytic Stability

Radiolabeling of Ibu-PSMA-02 with lutetium-177 was feasible at a molar activity up to 100 MBq/nmol with a radiochemical purity ≥98%. [^177^Lu]Lu-Ibu-PSMA-01 and [^177^Lu]Lu-PSMA-617 were obtained with a radiochemical purity ≥96%. All three radioligands were used for in vitro and in vivo experiments without further purification steps (Supplementary Materials, Figure S1).

At a high activity concentration (500 MBq/mL), radiolytic degradation of [^177^Lu]Lu-Ibu-PSMA-02 was observed, however, >84% radioligand were still intact after a 24 h-incubation period. Radiolysis of [^177^Lu]Lu-Ibu-PSMA-01 and [^177^Lu]Lu-PSMA-617 was more pronounced, resulting in <10% intact product under the same conditions. Radiolysis of all three radioligands was, however, entirely prevented in the presence of l-ascorbic acid, resulting in >96% intact radioligand after 24 h incubation (Supplementary Materials, Figure S2).

### 2.3. In Vitro Evaluation 

#### 2.3.1. Determination of the *n*-Octanol/PBS Distribution Coefficient

The *n*-octanol/PBS distribution coefficient (logD value) of [^177^Lu]Lu-Ibu-PSMA-02 was determined as −2.43 ± 0.01. This value was in the same range as the previously published logD value of [^177^Lu]Lu-Ibu-PSMA-01 (−2.18 ± 0.07) [17], but higher than the logD value of [^177^Lu]Lu-PSMA-617 (−4.38 ± 0.01) [11].

#### 2.3.2. Albumin-Binding Properties

**Figure 2:**
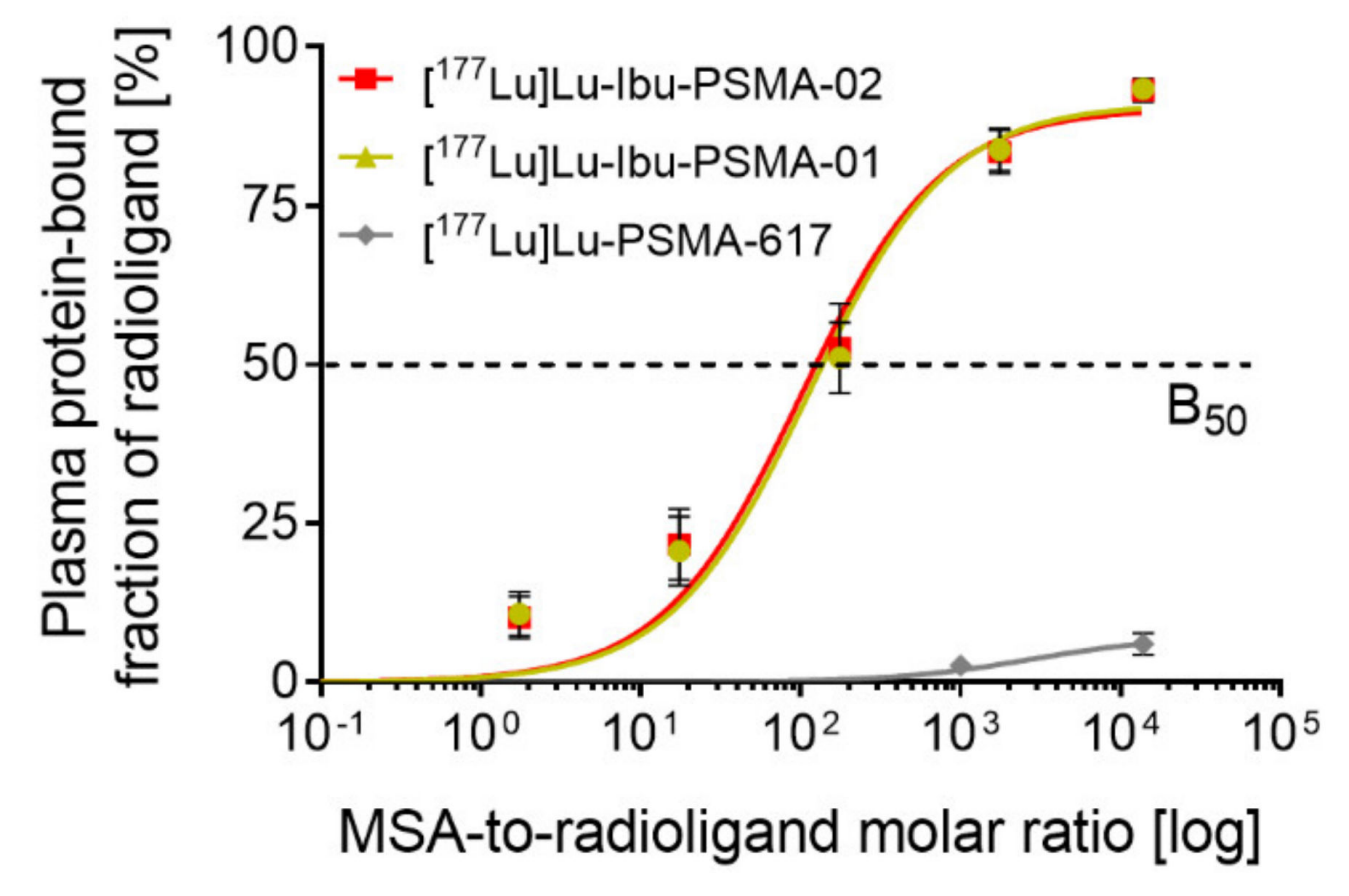
Data from ultrafiltration assays of [^177^Lu]Lu-Ibu-PSMA-02 in comparison to [^177^Lu]Lu-Ibu-PSMA-01 and [^177^Lu]Lu-PSMA-617 (*n* = 4). The data were fitted with a one-site specific binding curve to calculate the B_50_ values. MSA: Mouse serum albumin. Data of [^177^Lu]Lu-PSMA-617 were adapted with permission from Umbricht et al. 2018 Mol Pharm 15:2297 [16]. Copyright 2020 American Chemical Society.

[^177^Lu]Lu-Ibu-PSMA-02 and [^177^Lu]Lu-Ibu-PSMA-01 showed affinity to mouse plasma proteins with similar half-maximum binding (B_50_ values: ~79 and ~96, respectively), which was in clear contrast to [^177^Lu]Lu-PSMA-617 that showed negligible binding to mouse plasma proteins (Figure 2). Similarly, [^177^Lu]Lu-Ibu-PSMA-02 and [^177^Lu]Lu-Ibu-PSMA-01 showed increased binding to human plasma proteins in general and to human serum albumin in particular (Supplementary Materials, Figure S3).

#### 2.3.3. Cell Uptake and Internalization

**Figure 3:**
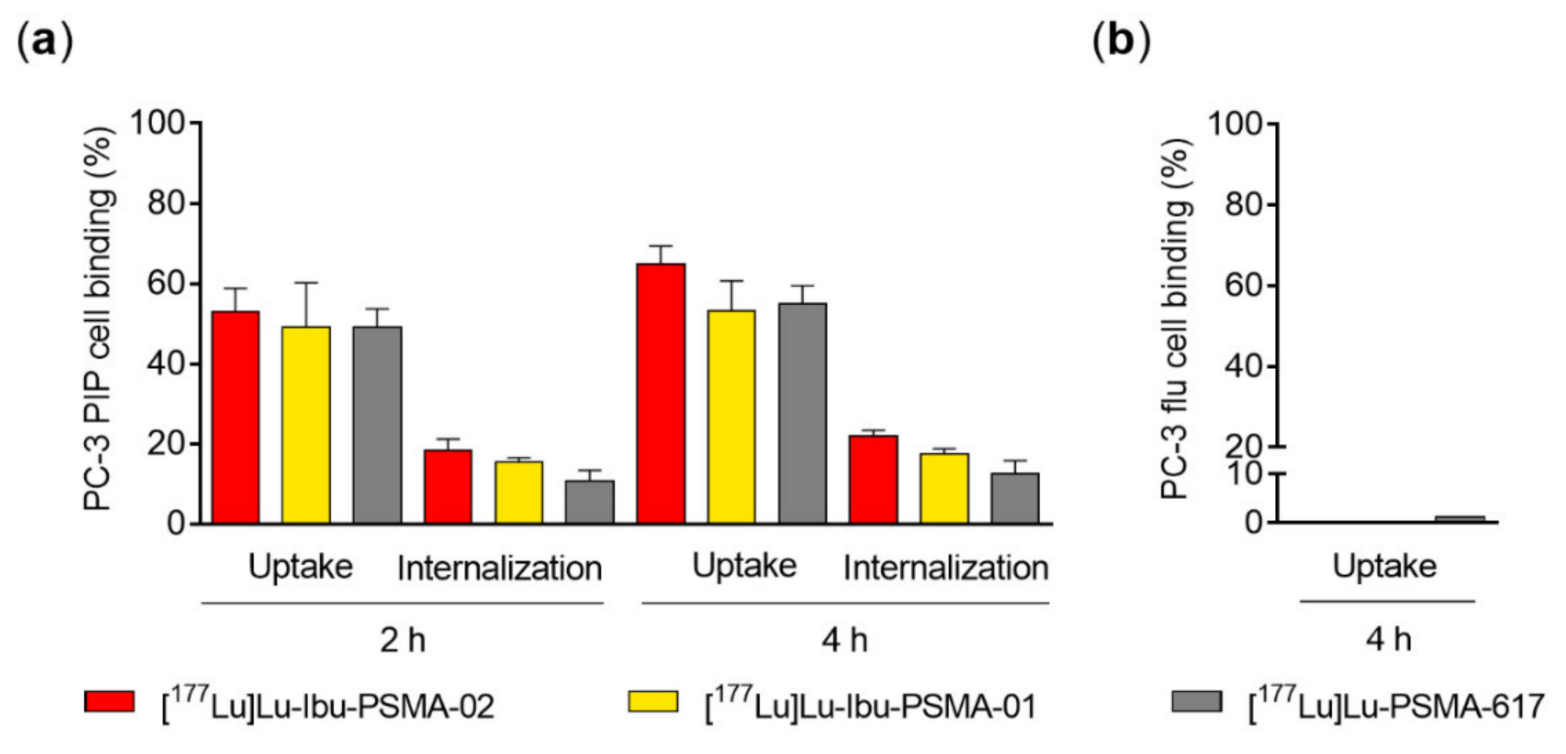
Uptake and internalization of [^177^Lu]Lu-Ibu-PSMA-02 in comparison to [^177^Lu]Lu-Ibu-PSMA-01 [17] and [^177^Lu]Lu-PSMA-617 [11]. (**a**) Data obtained in PSMA-positive PC-3 PIP cells (*n* = 3). (**b**) Data obtained in PSMA-negative PC-3 flu cells (*n* = 3). Data of [^177^Lu]Lu-PSMA-617 were adapted with permission from Benešová et al. 2018 Mol Pharm 15: 934. Copyright 2020 American Chemical Society [11]. Data of [^177^Lu]Lu-Ibu-PSMA-01 were adapted from Deberle and Benešová et al. 2020 Theranostics 10:1678 [17].

The uptake of [^177^Lu]Lu-Ibu-PSMA-02 in PC-3 PIP tumor cells (53% ± 6% and 65% ± 5%) was in the same range as for [^177^Lu]Lu-Ibu-PSMA-01 (49% ± 12% and 53% ± 8%) and [^177^Lu]Lu-PSMA-617 (49% ± 5% and 55% ± 5%) after a 2- and 4 h-incubation period, respectively (Figure 3a). The internalized fraction of [^177^Lu]Lu-Ibu-PSMA-02 (18% ± 3% and 22% ± 2%) was similar to [^177^Lu]Lu-Ibu-PSMA-01 (16%−17%) but higher than for [^177^Lu]Lu-PSMA-617 (11%−13%) after 2 and 4 h incubation (Figure 3a). The uptake of [^177^Lu]Lu-Ibu-PSMA-02 in PSMA-negative PC-3 flu tumor cells was <0.1% after 4 h incubation, which indicated PSMA-specific cell uptake in PC-3 PIP cells (Figure 3b). The same results were previously reported for [^177^Lu]Lu-Ibu-PSMA-01 [17] and [^177^Lu]Lu-PSMA-617 [11].

#### 2.3.4. Determination of PSMA-binding Affinity

The PSMA-binding affinity of [^177^Lu]Lu-Ibu-PSMA-02, determined using PC-3 PIP tumor cells, was in the same range (K_D_ = 40 ± 15 nM) as the affinity of [^177^Lu]Lu-Ibu-PSMA-01 (K_D_ = 24 ± 7 nM; *p* > 0.05) [17]. The binding affinity of [^177^Lu]Lu-Ibu-PSMA-02 was, however, significantly lower (*p* < 0.05) than the affinity of [^177^Lu]Lu-PSMA-617 (K_D_ = 13 ± 1 nM) [11].

### 2.4. Biodistribution Studies

[^177^Lu]Lu-Ibu-PSMA-02 showed high blood activity levels at 1 h p.i. (29% ± 4% injected activity per gram (IA/g)), which decreased to 7.1% ± 0.7% IA/g at 4 h and <1% IA/g 24 h after injection. The PC-3 PIP tumor uptake of [^177^Lu]Lu-Ibu-PSMA-02 was high already 1 h after injection (63% ± 8% IA/g) and further increased over the following 24 h (133% ± 15% IA/g; 24 h p.i.). Clearance of activity from tumor tissue was slow, which resulted in 56% ± 9% and 24% ± 3% IA/g retained activity in the tumor four and eight days after injection. Uptake of [^177^Lu]Lu-Ibu-PSMA-02 into PSMA-negative PC-3 flu tumors was below blood levels, confirming the PSMA-mediated uptake in PC-3 PIP tumors as was also the case for the other, previously investigated radioligands [11,16,17]. 

The kidney uptake of [^177^Lu]Lu-Ibu-PSMA-02 was high (115% ± 15% IA/g) 1 h after injection, but renal clearance was fast, resulting in activity levels of 27% ± 2% IA/g and 10% ± 2% IA/g at 4 and 24 h p.i., respectively. Accumulation in the kidneys was <4% IA/g at 48 h after injection of [^177^Lu]Lu-Ibu-PSMA-02. A high accumulation of activity was also observed in the liver at early timepoints (17% ± 4% IA/g at 1 h p.i. and 7.1% ± 0.5% IA/g at 4 h p.i.), but the fast clearance resulted in a low activity retention in the liver at later timepoints (<1% at 24 h p.i.). 

**Figure 4:**
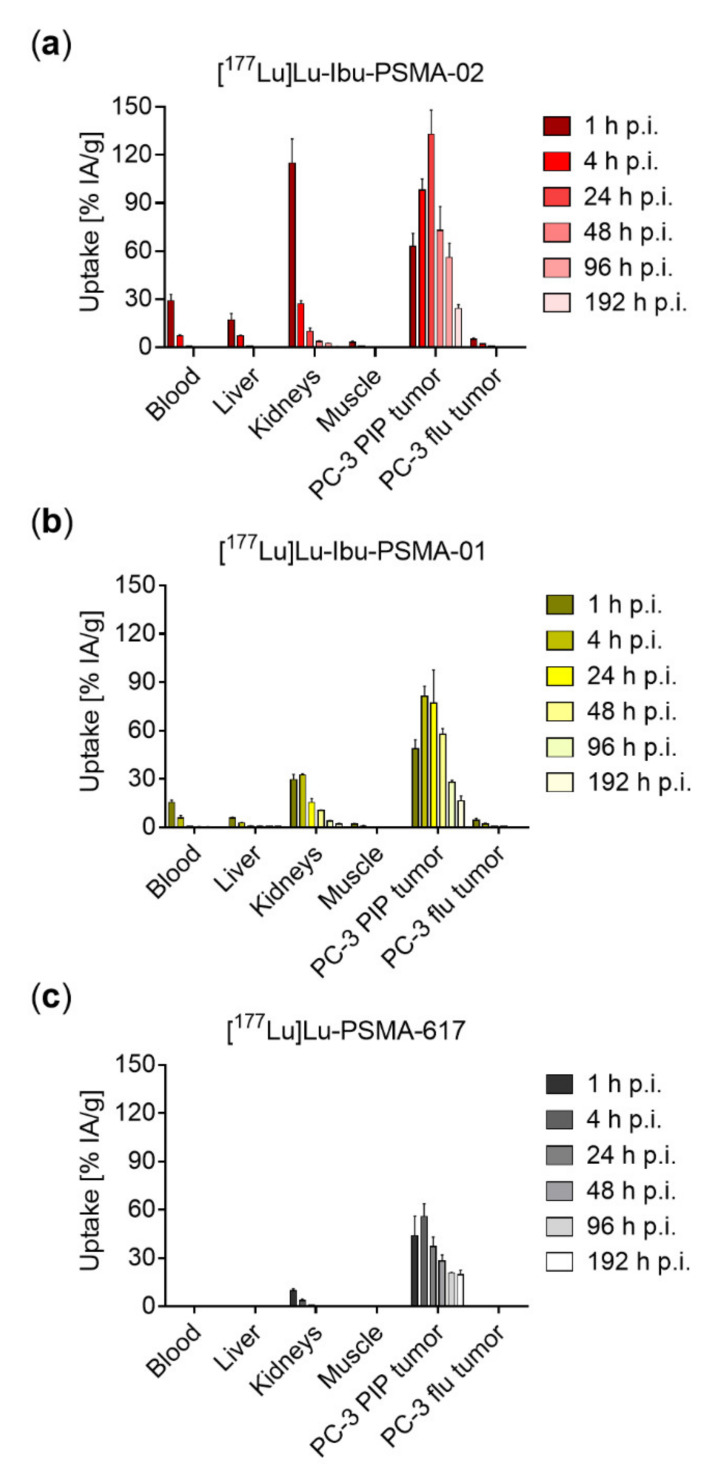
Graphs showing decay-corrected biodistribution data of [^177^Lu]Lu-Ibu-PSMA-02, [^177^Lu]Lu-Ibu-PSMA-01 and [^177^Lu]Lu-PSMA-617 obtained in PC-3 PIP/flu tumor-bearing mice. (**a**) Biodistribution data of [^177^Lu]Lu-Ibu-PSMA-02; (**b**) Previously published biodistribution data of [^177^Lu]Lu-Ibu-PSMA-01, adapted from Deberle and Benešová et al. 2020 Theranostics 10:1678 [17]; (**c**) Previously published biodistribution data of [^177^Lu]Lu-PSMA-617, adapted with permission from Benešová et al. 2018 Mol Pharm 15:934. Copyright 2020 American Chemical Society [11].

Overall, the uptake of [^177^Lu]Lu-Ibu-PSMA-02 in the tumor was significantly higher (*p* < 0.05) and longer retained as compared to [^177^Lu]Lu-Ibu-PSMA-01. Accumulation in non-targeted organs and tissue was also higher in the case of [^177^Lu]Lu-Ibu-PSMA-02 at early timepoints. The fast clearance of [^177^Lu]Lu-Ibu-PSMA-02 resulted, however, in similar or even lower retention of activity in kidneys at timepoints later than 1 h p.i. and in the liver later than 4 h p.i. [17]. Due to the presence of an albumin-binding entity and the resulting increased blood circulation time, maximum tumor uptake of [^177^Lu]Lu-Ibu-PSMA-02 was reached at 24 h p.i. and was ~2.5-fold higher than the maximum tumor uptake of [^177^Lu]Lu-PSMA-617 which was reached at 4 h p.i. Off-target accumulation of [^177^Lu]Lu-Ibu-PSMA-02 as well as of [^177^Lu]Lu-Ibu-PSMA-01 was, however, also higher than for [^177^Lu]Lu-PSMA-617 at all investigated timepoints (Figure 4, Supplementary Materials, Tables S1‒S3).

High blood pool activity 1 h after injection of [^177^Lu]Lu-Ibu-PSMA-02 resulted in a low tumor-to-blood ratio (2.2 ± 0.1), which increased significantly over time due to the efficient blood clearance. Tumor-to-blood ratios were in the range of 170−220 between 24 and 96 h after injection of [^177^Lu]Lu-Ibu-PSMA-02 and reached a value of 918 ± 80 after eight days. The tumor-to-kidney ratio of [^177^Lu]Lu-Ibu-PSMA-02 was low at 1 h p.i. (0.56 ± 0.09), but increased significantly with time to a tumor-to-kidney ratio >50 eight days after injection. A similar time course was observed for the tumor-to-liver ratios.

**Figure 5:**
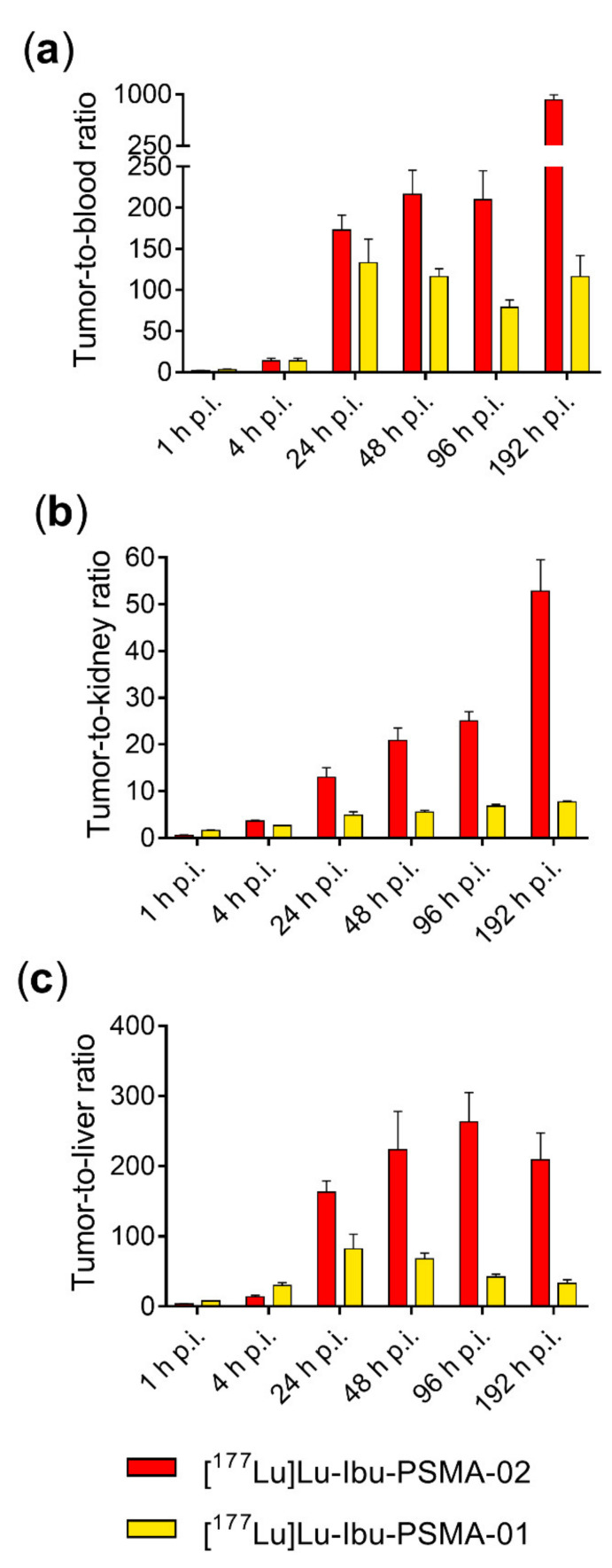
(**a**) Tumor-to-blood ratios; (**b**) tumor-to-kidney ratios and (**c**) tumor-to-liver ratios at 1, 4, 24, 48, 96 and 192 h after injection of [^177^Lu]Lu-Ibu-PSMA-02 in comparison to [^177^Lu]Lu-Ibu-PSMA-01 [17].

Overall, the tumor-to-background ratios of [^177^Lu]Lu-Ibu-PSMA-02 were relatively low at early timepoints, but increased significantly over time. As a result, the tumor-to-blood, tumor-to-kidney and tumor-to-liver ratios of [^177^Lu]Lu-Ibu-PSMA-02 were higher than those of [^177^Lu]Lu-Ibu-PSMA-01 at timepoints later than 4 h p.i. (Figure 5) [17]. Due to the lack of a designated albumin binder and thus extremely fast clearance from background organs, tumor-to-background ratios of [^177^Lu]Lu-PSMA-617 were higher than those of the albumin-binding radioligands at all investigated timepoints (Supplementary Materials, Figure S4) [11].

Calculation of the area under the curve (AUC) values of [^177^Lu]Lu-Ibu-PSMA-02 revealed a 1.6-fold increased AUC tumor value and a 1.2-fold increased AUC blood value over the course of 192 h in comparison to [^177^Lu]Lu-Ibu-PSMA-01. As a result of the increased tumor uptake, the tumor-to-blood AUC ratio of [^177^Lu]Lu-Ibu-PSMA-02 was 1.4-fold higher than that of [^177^Lu]Lu-Ibu-PSMA-01 (Supplementary Materials, Figure S5, Table S4).

### 2.5. Dosimetric Calculations

**Figure 6:**
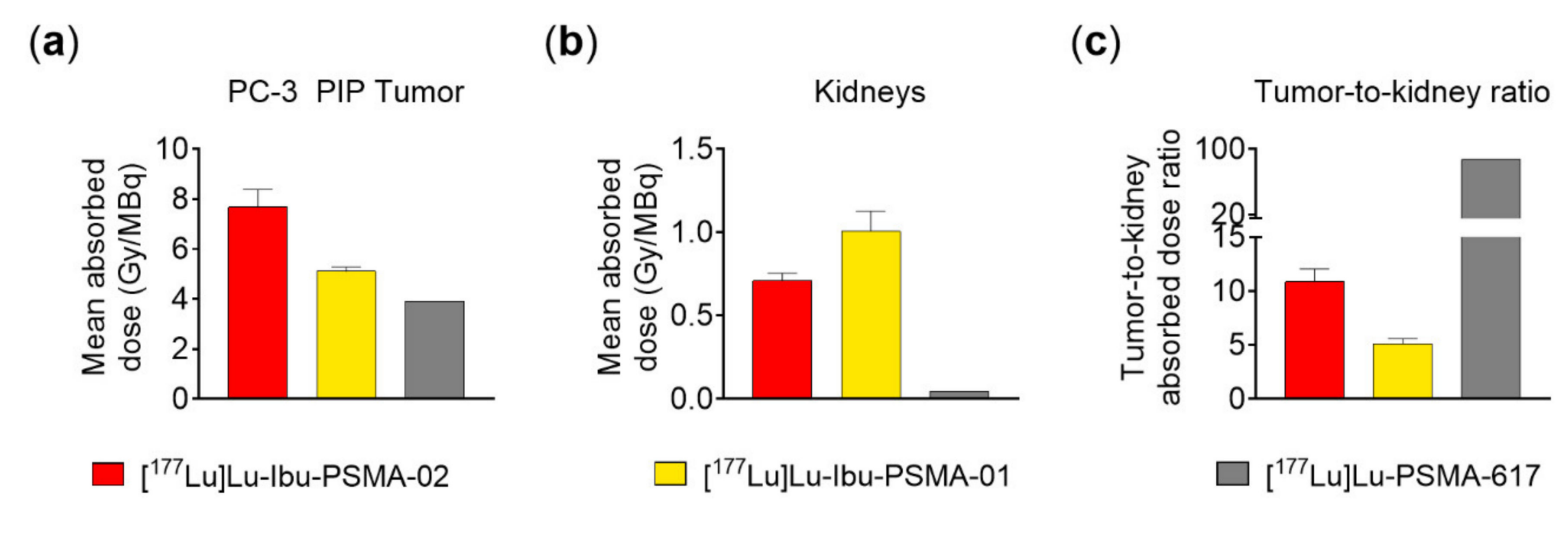
The graphs show dosimetry results of [^177^Lu]Lu-Ibu-PSMA-02 in comparison to [^177^Lu]Lu-Ibu-PSMA-01 and [^177^Lu]Lu-PSMA-617. (**a**) Values representing the mean absorbed doses of the PC-3 PIP tumor; (**b**) mean absorbed kidney doses, and (**c**) the tumor-to-kidney absorbed dose ratios for [^177^Lu]Lu-Ibu-PSMA-02, [^177^Lu]Lu-Ibu-PSMA-01 and [^177^Lu]Lu-PSMA-617 [19]. Data for [^177^Lu]Lu-PSMA-617 adapted from Müller et al. 2019 Eur J Nucl Med Mol Imaging 46: 1919 [19].

High accumulation and retention of [^177^Lu]Lu-Ibu-PSMA-02 in the tumor led to a mean absorbed tumor dose of 7.7 ± 0.8 Gy/MBq. The mean specific absorbed dose to the kidneys was 0.71 ± 0.05 Gy/MBq, resulting in a tumor-to-kidney absorbed dose ratio of 11 ± 2. In contrast to [^177^Lu]Lu-Ibu-PSMA-02, the mean absorbed tumor dose of [^177^Lu]Lu-Ibu-PSMA-01 was lower (5.1 ± 0.2 Gy/MBq) and the mean absorbed dose to the kidneys higher (1.0 ± 0.2 Gy/MBq). This led to a lower tumor-to-kidney absorbed dose ratio of 5.1 ± 0.5. Mean absorbed doses for both the tumor and the kidneys were clearly lower in the case of [^177^Lu]Lu-PSMA-617 (3.90 and 0.05 Gy/MBq, respectively) and, the resulting tumor-to-kidney dose ratio (~87), therefore, much higher as compared to the albumin-binding radioligands (Figure 6) [19]. 

### 2.6. SPECT/CT Imaging

**Figure 7:**
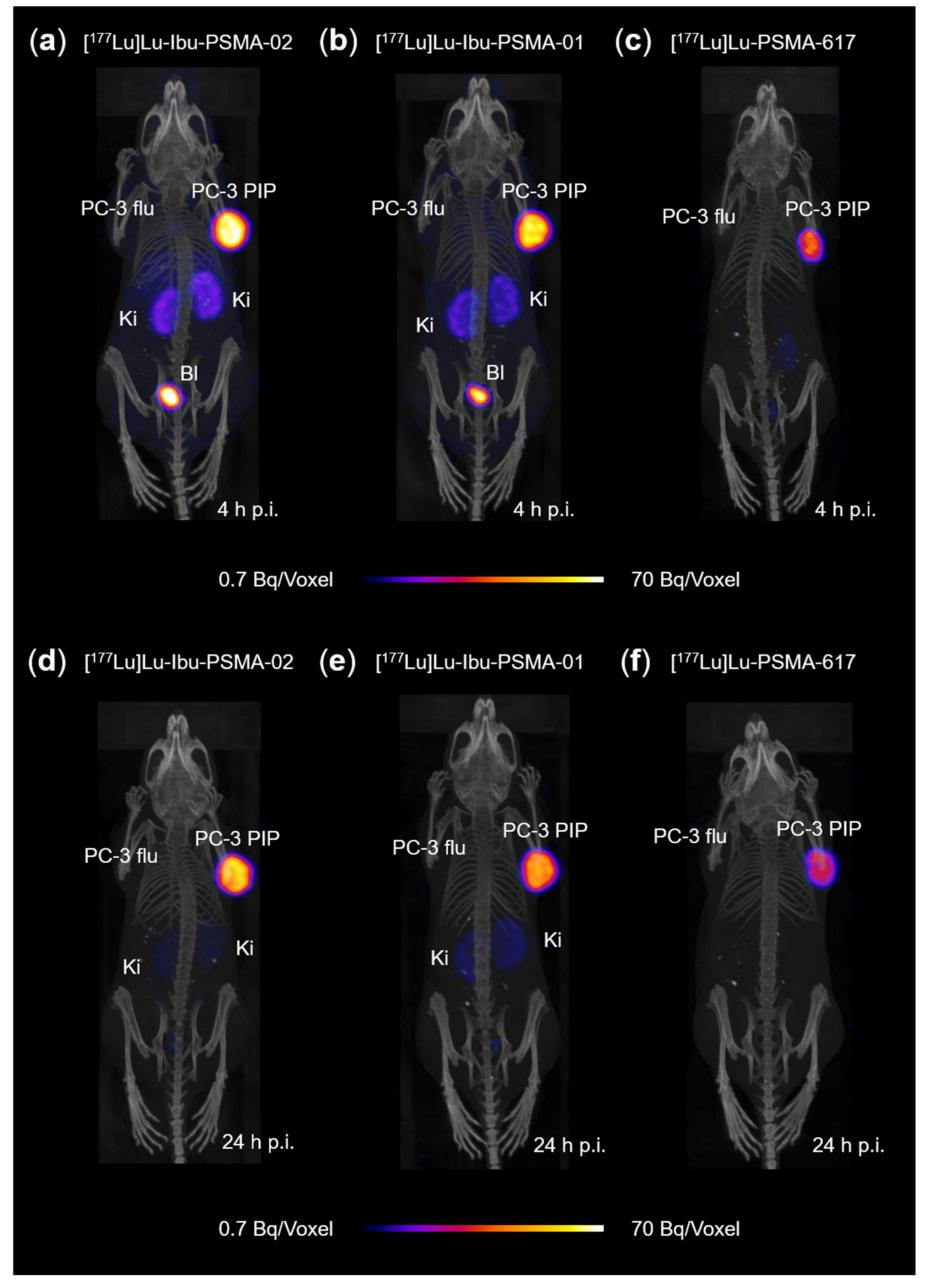
SPECT/CT images obtained 4 h and 24 h after injection of the PSMA radioligands and shown as maximum intensity projections (MIPs); (**a**) MIP of [^177^Lu]Lu-Ibu-PSMA-02 4 h p.i.; (**b**) MIP of [^177^Lu]Lu-Ibu-PSMA-01 4 h p.i.; (**c**) MIP of [^177^Lu]Lu-PSMA-617 4 h p.i.; (**d**) MIP of [^177^Lu]Lu-Ibu-PSMA-02 24 h p.i.; (**e**) MIP of [^177^Lu]Lu-Ibu-PSMA-01 24 h p.i. and (**f**) MIP of [^177^Lu]Lu-PSMA-617 24 h p.i.; PC-3 PIP = PSMA-positive tumor xenograft; PC-3 flu = PSMA-negative tumor xenograft; Ki = kidney; Bl = urinary bladder.

The SPECT/CT images of mice visualized the PC-3 PIP tumor xenograft (right shoulder), in which [^177^Lu]Lu-Ibu-PSMA-02 accumulated to a high extent, whereas in the PC-3 flu tumors (left shoulder) accumulation of activity was not observed (Figure 7). At the 4-h timepoint, some activity was also seen in the kidneys as well as in the urinary bladder as a result of the radioligand’s renal excretion. At the 24-h timepoint, activity was almost exclusively visualized in the PC-3 PIP tumor. At both investigated timepoints, the tumor uptake of [^177^Lu]Lu-Ibu-PSMA-02 was slightly higher than the uptake of [^177^Lu]Lu-Ibu-PSMA-01 and considerably higher than the uptake of [^177^Lu]Lu-PSMA-617 (Figure 7). 

## 3. Discussion

In this study, the impact of a small structural change on the in vitro and in vivo behavior of a PSMA radioligand was investigated. The newly developed radioligand [^177^Lu]Lu-Ibu-PSMA-02 differed from [^177^Lu]Lu-Ibu-PSMA-01 by two carbon atoms in the linker entity which connected ibuprofen with the radioligand’s backbone.

The synthesis of Ibu-PSMA-02 was performed in analogy to the previously published synthesis of Ibu-PSMA-01 [17] by means of a resin-immobilized precursor containing a diaminobutyric acid entity. Ibu-PSMA-02 was obtained after a single purification step in high purity. This was indirectly confirmed by the feasibility of radiolabeling at a molar activity up to 100 MBq/nmol with a radiochemical purity of >98%. Interestingly, [^177^Lu]Lu-Ibu-PSMA-02 was considerably more stable than [^177^Lu]Lu-PSMA-617 and other previously synthesized ibuprofen-derivatized PSMA radioligands [17]. In this regard, [^177^Lu]Lu-Ibu-PSMA-02 rather resembled [^177^Lu]Lu-PSMA-ALB-56 containing a *p*-tolyl entity as stabilizing albumin binder [16]. This result showed that the radiolytic stability of a radioligand can be effectively influenced by small structural changes. Similarly, Kelly et al. demonstrated that the structure of the linker entity might have a significant impact on the stability of PSMA radioligands [18].

Evaluation of the concentration-dependent affinities of both albumin-binding radioligands to mouse plasma proteins showed that the small structural changes in the connecting entity between ibuprofen and the PSMA ligand’s backbone did not have an impact on the binding affinity. In analogy, binding to human plasma proteins and to human serum albumin showed similar results between the radioligands (Supplementary Materials, Figure S3). Notably, the B_50_ values of [^177^Lu]Lu-Ibu-PSMA-02 and [^177^Lu]Lu-Ibu-PSMA-01 were similar to that of [^177^Lu]Lu-PSMA-ALB-56 containing a *p*-tolyl-based albumin binder [16], which indicates that ibuprofen and the *p*-tolyl entity exhibit comparable affinities towards plasma proteins. It has to be mentioned, however, that the experiments regarding the plasma protein-binding properties of [^177^Lu]Lu-PSMA-ALB-56 were performed using a slightly different method as compared to those for [^177^Lu]Lu-Ibu-PSMA-02 and [^177^Lu]Lu-Ibu-PSMA-01.

Excretion of the radioligand from the whole body of non-tumor-bearing mice showed increased retention of [^177^Lu]Lu-Ibu-PSMA-02 in comparison to [^177^Lu]Lu-PSMA-617 (Supplementary Materials, Figure S6). Comparison with [^177^Lu]Lu-Ibu-PSMA-01 showed a similar excretion profile, although at later timepoints, [^177^Lu]Lu-Ibu-PSMA-02 was cleared somewhat faster. 

Even though the structural differences between Ibu-PSMA-02 and Ibu-PSMA-01 were small, the tissue distribution profile of [^177^Lu]Lu-Ibu-PSMA-02 in PC-3 PIP tumor-bearing mice was different to the profile previously obtained with [^177^Lu]Lu-Ibu-PSMA-01 [17]. Stability studies confirmed that both radioligands were entirely stable under the conditions of radioligand preparation for biodistribution studies (data not shown). These results ensured that the radioligand solutions injected into the mice did not contain any degradation products, which could have influenced the tissue distribution. 

Mice injected with [^177^Lu]Lu-Ibu-PSMA-02 showed higher accumulation of activity in PC-3 PIP tumors as compared to [^177^Lu]Lu-Ibu-PSMA-01 and other ibuprofen-containing PSMA radioligands [17]. This may have been a consequence of the increased activity level in the blood pool of [^177^Lu]Lu-Ibu-PSMA-02 at early timepoints. Obviously, in vitro albumin-binding properties of a radioligand under equilibrium conditions cannot be considered as the only predictor of its blood retention time, and consequently, tissue distribution profile. The somewhat higher retention of [^177^Lu]Lu-Ibu-PSMA-02 in the blood resulted, however, also in higher uptake in non-targeted tissues and organs, particularly in the kidneys. Despite increased off-target accumulation of [^177^Lu]Lu-Ibu-PSMA-02 at early timepoints after injection, the tumor-to-kidney and tumor-to-liver ratios at later timepoints were higher than for other albumin-binding radioligands evaluated under the same experimental conditions (Supplementary Materials, Figure S4) [11,17]. The high PSMA expression level of PC-3 PIP tumors does, however, not allow a direct comparison with other radioligands that were evaluated in LNCaP tumor-bearing mice [18,20] since LNCaP tumors express PSMA at considerably lower levels [21]. Moreover, the injected molar amount of the radioligand may have a critical impact on its tissue distribution profile as well. In agreement with biodistribution data, the SPECT/CT images showed increased accumulation of [^177^Lu]Lu-Ibu-PSMA-02 in the PC-3 PIP tumor as compared to the uptake of [^177^Lu]Lu-Ibu-PSMA-01 and [^177^Lu]Lu-PSMA-617, but also higher uptake in the kidneys at 4 h p.i. Intense activity signals were also found in the urinary bladder for both albumin-binding radioligands. This can be ascribed to residual activity after emptying the urinary bladder before acquisition of the SPECT/CT image, and from the ongoing urine formation during the scan.

Dosimetry calculations confirmed the superior mean absorbed dose to the tumor of [^177^Lu]Lu-Ibu-PSMA-02 in comparison to [^177^Lu]Lu-Ibu-PSMA-01 and [^177^Lu]Lu-PSMA-617. It can thus be speculated that [^177^Lu]Lu-Ibu-PSMA-02 would exhibit higher therapeutic efficacy as compared to the clinically used [^177^Lu]Lu-PSMA-617. Moreover, the tumor-to-kidney dose ratio of [^177^Lu]Lu-Ibu-PSMA-02 was significantly higher than for [^177^Lu]Lu-Ibu-PSMA-01, which is a favorable characteristic in view of a therapeutic application. The tumor-to-kidney dose ratio of [^177^Lu]Lu-PSMA-617 was higher than that of the albumin-binding radioligands, but the absolute value of the mean absorbed tumor dose of [^177^Lu]Lu-PSMA-617 remained relatively low in comparison to the new radioligands.

The increased blood circulation of albumin-binding PSMA radioligands certainly poses the concern of an increased bone marrow toxicity. The absorbed dose to the bone marrow is, however, not easily assessable. Based on AUC calculations, the tumor-to-blood AUC ratio of [^177^Lu]Lu-Ibu-PSMA-02 was, however, higher than for [^177^Lu]Lu-Ibu-PSMA-01, which would certainly be favorable in view of a therapeutic application (Supplementary Materials, Figure S5, Table S4).

Additional in-depth studies will be necessary in order to fully understand the observed discrepancies among the biodistribution profiles of [^177^Lu]Lu-Ibu-PSMA-02 and [^177^Lu]Lu-Ibu-PSMA-01.

## 4. Material and Methods

### 4.1. Synthesis of Ibu-PSMA-02

**Scheme 1:**
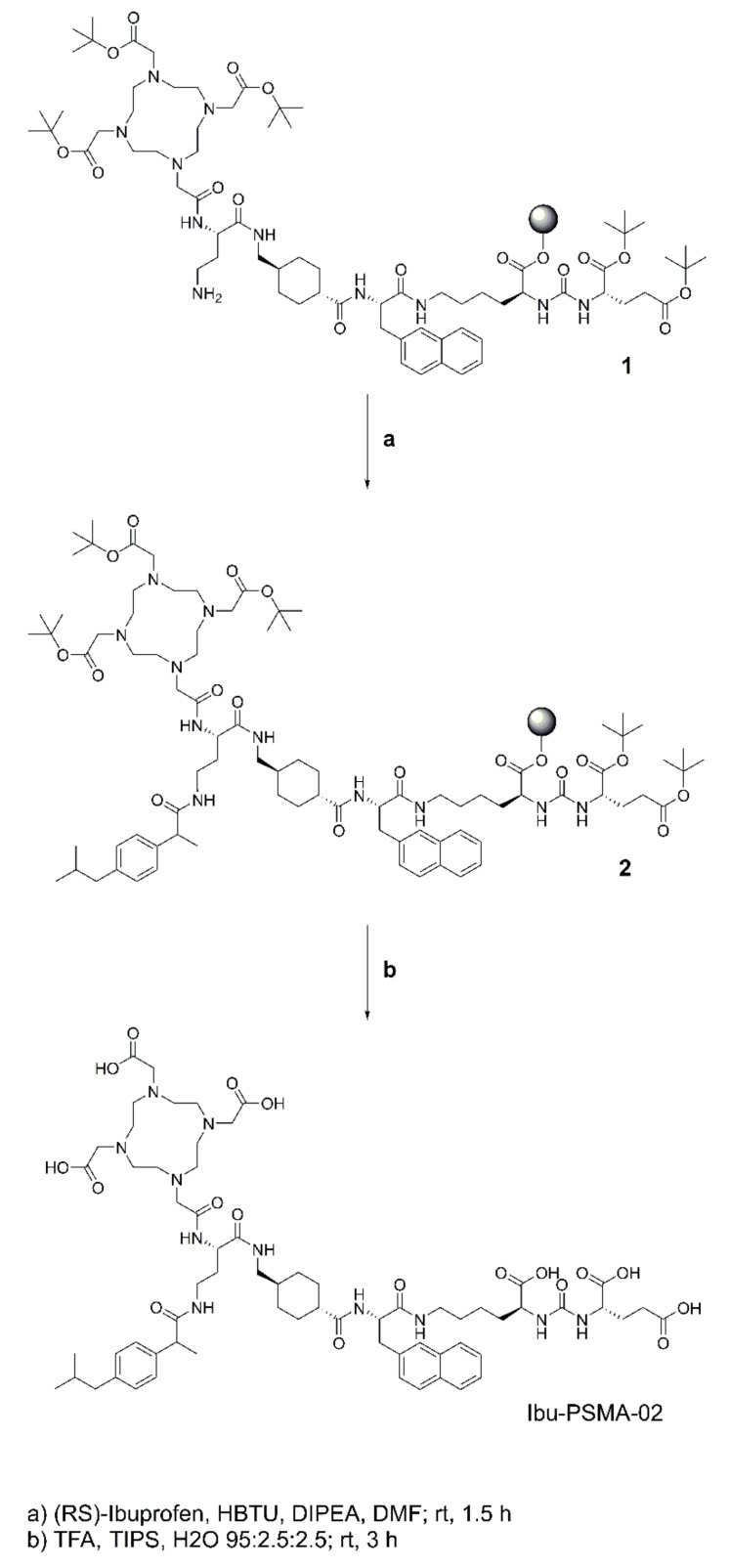
Synthesis of Ibu-PSMA-02 based on precursor **1**.

In analogy to previously synthesized ibuprofen-derivatized PSMA ligands [17], Ibu-PSMA-02 was synthesized via a solid-phase platform. The synthesis of Ibu-PSMA-02 was based on precursor **1**, which was prepared in analogy to the methods previously described (Supplementary Materials, Scheme S1) [6,11]. Instead of a l-lysine residue, l-diaminobutyric acid was introduced as a linker to conjugate (*RS*)-ibuprofen with the PSMA ligand’s backbone. (*RS*)-Ibuprofen was coupled to precursor **1** to obtain compound **2** in the same manner as previously reported by Deberle and Benešová et al. (Supplementary Materials) [17]. Ibu-PSMA-02 was obtained after cleavage of the compound from the resin, removal of the protecting groups and HPLC purification of the crude product (Scheme 1). The isolated, pure PSMA ligand was characterized by analytical HPLC and MALDI-TOF-MS (Bruker UltraFlex II, Billerica, MA, U.S.A.) (Supplementary Materials).

### 4.2. Radiolabeling and Radiolytic Stability 

Ibu-PSMA-02 (1 mM stock solution) was labeled with lutetium-177 (no-carrier-added lutetium-177 in 0.04 M HCl; ITM Medical Isotopes GmbH, Germany) at molar activities of 5–100 MBq/nmol at pH ~4.5 as previously reported (Supplementary Materials) [16]. The reaction mixture was incubated for 10 min at 95 °C, followed by quality control using HPLC as previously reported (Supplementary Materials) [11,16]. Ibu-PSMA-01, which was previously developed in our group [17], and PSMA-617 (ABX GmbH, Radeberg, Germany) were radiolabeled under the same conditions.

The radiolytic stability of [^177^Lu]Lu-Ibu-PSMA-02 was assessed in three independent experiments. [^177^Lu]Lu-Ibu-PSMA-02 (50 MBq/nmol) was diluted with saline to an activity concentration of 250 MBq/500 µL and incubated at room temperature with or without the addition of l-ascorbic acid (3 mg) for a total period of 24 h. HPLC was performed after 1, 4 and 24 h to determine the radioligand’s integrity as previously reported (Supplementary Materials) [17]. 

### 4.3. Determination of LogD Values

The in vitro evaluation of [^177^Lu]Lu-Ibu-PSMA-02 was carried out as previously reported for other ibuprofen-modified PSMA radioligands [11,17]. The *n*-octanol/PBS distribution coefficient (LogD) of [^177^Lu]Lu-Ibu-PSMA-02 (50 MBq/nmol) was determined in a mixture of *n*-octanol and PBS, pH 7.4, by a shake-flask method using liquid–liquid extraction followed by phase separation and measurement of activity in both phases using a γ-counter (Perkin Elmer Wallac Wizard 1480). The distribution coefficient was calculated as the logarithm of the ratio of counts per minute (cpm) measured in the *n*-octanol phase relative to the cpm measured in the PBS phase. Three experiments were performed in quintuplicate.

### 4.4. Albumin-Binding Properties

The binding of [^177^Lu]Lu-Ibu-PSMA-02 and [^177^Lu]Lu-Ibu-PSMA-01 to mouse plasma proteins was evaluated by incubating a fixed amount of radioligand with different concentrations of mouse plasma and followed by determination of the percentage of bound radioligand using an ultrafiltration method. Mouse plasma was diluted to obtain a mouse serum albumin (MSA) concentration of 0.07 to 550 μM in PBS pH 7.4. A fixed amount of the radioligand (50 MBq/nmol; 15 µL, ~300 kBq, 0.006 nmol,) was added to 150 µL of the mouse plasma dilutions, briefly vortexed and incubated for 30 min at 37 °C. Afterwards, each sample of mouse plasma-radioligand mixture was cooled on ice followed by addition of 300 µL ice-cold PBS. The samples were transferred to Amicon centrifugal filters (cut-off of 10 kDa), which ensured the retention of MSA (~67 kDa), and centrifuged (rcf of 14,000) for 30 min at 4 °C. The inserts of the filter devices were inverted and centrifuged (rcf of 200) for 3 min to recover the protein fraction. The activity of the protein fraction (A_protein_) and the activity in the filtrate and in the filter unit were measured separately in a γ-counter. The total activity (A_total_) was determined as the sum of counted activity of the protein fraction (A_protein_), the filtrate and the filter unit. The percentage of radioligand bound to mouse plasma proteins was calculated as (bound radioligand = A_protein_/A_total_ *100).

The results were presented as average ± standard deviation (SD) of four independent experiments. The percentage of radioligand bound to mouse plasma (A_protein_) was plotted against the MSA-to-radioligand molar ratios and fitted with a nonlinear regression curve (one site, specific binding) using GraphPad Prism software (version 7, San Diego, CA, U.S.A.) to obtain the half-maximum binding (B_50_). Control experiments were performed by incubating the radioligands with PBS only followed by filtration which resulted in ≤10% of unspecifically retained radioligand in the filter. The binding of [^177^Lu]Lu-PSMA-617 to mouse plasma proteins was previously determined by Umbricht et al. using a similar procedure but a different ultrafiltration device [16].

Additionally, the radioligands were incubated in human plasma and in 700 µM human serum albumin (HSA) diluted in PBS to confirm the binding also to human plasma proteins and albumin, respectively (Supplementary Materials). 

### 4.5. Cell Culture and Cell Experiments

Sublines of the androgen-independent PC-3 human prostate cancer cell line, PSMA-positive PC-3 PIP and PSMA-negative PC-3 flu cells, were kindly provided by Prof. Dr. Martin Pomper (Johns Hopkins University School of Medicine, Baltimore, MD, U.S.A.) (Supplementary Materials). The tumor cells were cultured in RPMI-1640 cell culture medium supplemented with 10% fetal calf serum, l-glutamine and antibiotics. Puromycin (2 µg/mL) was added to the cell cultures to maintain PSMA expression as previously reported [21].

Cell uptake and internalization studies of [^177^Lu]Lu-Ibu-PSMA-02 were performed as previously reported [11]. The radioligand was investigated in three experiments performed in six replicates with PC-3 PIP tumor cells and in triplicate with PC-3 flu tumor cells. The PSMA-binding affinity of [^177^Lu]Lu-Ibu-PSMA-02, presented as K_D_ value, was determined as previously reported [17]. Statistical significance of K_D_ values was assessed using a one-way ANOVA with a Tukeys’s multiple comparison post-test in GraphPad Prism software (version 7, San Diego, CA, U.S.A.). A *p*-value of <0.05 was considered statistically significant.

### 4.6. Tumor Mouse Model

All applicable international, national, and/or institutional guidelines for the care and use of animals were followed. In particular, all animal experiments were carried out according to the guidelines of Swiss Regulations for Animal Welfare. The preclinical studies were ethically approved by the Cantonal Committee of Animal Experimentation and permitted by the responsible cantonal authorities (license N° 75668).

Mice were obtained from Charles River Laboratories, Sulzfeld, Germany, at the age of 5–6 weeks. Female, athymic nude BALB/c mice were subcutaneously inoculated with PSMA-positive PC-3 PIP cells (6 × 10^6^ cells in 100 μL Hank’s balanced salt solution (HBSS)) on the right shoulder and with PSMA-negative PC-3 flu cells (5 × 10^6^ cells in 100 μL HBSS) on the left shoulder. Biodistribution and SPECT/CT imaging studies were performed about 2 weeks later, when the tumor sizes reached a volume of 80–300 mm^3^.

### 4.7. Biodistribution Study

[^177^Lu]Lu-Ibu-PSMA-02 (5 MBq, 1 nmol, 100 µL) was diluted in saline containing 0.05% bovine serum albumin (BSA) and injected in a lateral tail vein of the mice. Mice were euthanized at different timepoints after injection (p.i.) of the radioligand. Selected tissues and organs were collected, weighed and measured for activity using a γ-counter. The results were decay-corrected and listed as percentage of the injected activity per gram of tissue mass (% IA/g) (Figure 4, Supplementary Materials, Table S1). The values of the areas under the curve (AUCs) for the PC-3 PIP tumor and the blood were calculated based on non-decay-corrected biodistribution data (Supplementary Materials).

### 4.8. Dosimetric Calculations

Dosimetric calculations for [^177^Lu]Lu-Ibu-PSMA-02 and [^177^Lu]Lu-Ibu-PSMA-01 were performed based on non-decay-corrected biodistribution data. The cumulated activity was estimated by calculating the time-integrated activity concentration coefficients (TIACCs) and used for calculation of the mean specific absorbed dose (Gy/MBq) to the tumor xenografts and kidneys (Supplementary Materials). The absorbed fractions for the tumors and the kidneys were assessed by Monte Carlo simulations using PENELOPE [22]. Dosimetric calculations for [^177^Lu]Lu-PSMA-617 were previously performed with a different method as published by Müller et al. [19].

### 4.9. In Vivo SPECT/CT Imaging

SPECT/CT images were obtained using a dedicated small-animal SPECT/CT scanner (NanoSPECT/CT^TM^, Mediso Medical Imaging Systems, Budapest, Hungary) as previously reported [21]. [^177^Lu]Lu-Ibu-PSMA-02 (25 MBq, 1 nmol, 100 µL) diluted in saline containing 0.05% BSA was injected in a lateral tail vein of the mouse. SPECT scans of ~45 min duration were performed 4 and 24 h after injection of the radioligand followed by a CT of 7.5 min duration. During the in vivo scans, the mice were anesthetized with a mixture of isoflurane and oxygen. Reconstruction of the acquired data was performed using HiSPECT software (version 1.4.3049, Scivis GmbH, Göttingen, Germany). All images were prepared using *VivoQuant* post-processing software (version 3.5, inviCRO Imaging Services and Software, Boston, MA, U.S.A.). A Gauss post-reconstruction filter (FWHM = 1.0 mm) was applied to the images and the scale of radioactivity was set as indicated on the images (minimum value = 0.7 Bq/voxel to maximum value = 70 Bq/voxel).

## 5. Conclusions 

This study demonstrated that even subtle changes in the chemical structure may have a major impact on the pharmacokinetic properties of a PSMA radioligand, as exemplified by the characteristics of [^177^Lu]Lu-Ibu-PSMA-02 and [^177^Lu]Lu-Ibu-PSMA-01. [^177^Lu]Lu-Ibu-PSMA-02 comprising a shorter linker showed unprecedentedly high tumor uptake as a consequence of a relatively high blood pool activity at early timepoints after injection. Further investigations are warranted to better understand the relationship between the structure of ibuprofen-derivatized PSMA radioligands and their in vivo properties.

## Figures and Tables

**Table 1:** Analytical data of Ibu-PSMA-02 and Ibu-PSMA-01.

Compound	Chemical Formula	MW [g/mol]	*m*/*z*_calc_	*m*/*z*_meas_^1^	t_r_ [min]	Chemical Purity ^2^ [%]
Ibu-PSMA-02	C_66_H_95_N_11_O_18_	1330.55	1330.69	1330.69	10.4	>99
Ibu-PSMA-01^3^	C_68_H_99_N_11_O_18_	1358.60	1358.72	1358.72	9.9	>99

^1^*m*/*z*-peak in the unlabeled ligand obtained by MALDI-TOF-MS detected as [M + H]^+^; ^2^ Determined by analytical HPLC, λ = 254 nm; ^3^ ligand previously synthesized and published by Deberle and Benešová et al. 2020 [17].

## Supplementary Materials

The following are available online, HPLC Purification and Analysis of Ibu-PSMA-02; Radiolabeling, including Figure S1: HPLC chromatograms of radioligands obtained as a result of the quality control; Radiolytic Stability, including, Figure S2: Radiolytic stability of [^177^Lu]Lu-Ibu-PSMA-02, [^177^Lu]Lu-Ibu-PSMA-01 and [^177^Lu]Lu-PSMA-617; Albumin-Binding Properties, including, Figure S3: Plasma protein-binding properties of [^177^Lu]Lu-Ibu-PSMA-02 in comparison to [^177^Lu]Lu-Ibu-PSMA-01 and [^177^Lu]Lu-PSMA-617; Biodistribution, including Tables S1–S3: Decay-corrected biodistribution data of [^177^Lu]Lu-Ibu-PSMA-02, [^177^Lu]Lu-Ibu-PSMA-01 and [^177^Lu]Lu-PSMA-617 obtained in PC-3 PIP/flu tumor-bearing mice and Figure S4: Tumor-to-kidney ratios and tumor-to-liver ratios after injection of [^177^Lu]Lu-Ibu-PSMA-02 in comparison to [^177^Lu]Lu-Ibu-PSMA-01 and [^177^Lu]Lu-PSMA-617; Area under the Curve (AUC) Values and AUC Ratios, including Figure S5: Area under the curves (AUCs) of [^177^Lu]Lu-Ibu-PSMA-02 and [^177^Lu]Lu-Ibu-PSMA-01 and Table S4: Area under the curve (AUC) values and ratios of AUCs based on non-decay-corrected biodistribution data; Whole-Body-Activity Measurement, including Figure S6: Whole-body activity measured at various timepoints after injection of the radioligands; Synthesis of Ibu-PSMA-02, including Scheme S1: Solid-phase synthesis of precursor 1 used for the formation of Ibu-PSMA-02; Cell Culture; Dosimetric Calculations.
