# Supplementary material for: Albumin-Binding PSMA Radioligands: Impact of Minimal Structural Changes on the Tissue Distribution Profile
Source: Molecules. 2020 May 29;25(11):2542. doi: 10.3390/molecules25112542 (PMC7321364; doi:10.3390/molecules25112542)
Supplement: Supplementary file 1 [file molecules-25-02542-s001.pdf]

**SUPPLEMENTARY MATERIAL**

# Albumin-binding PSMA Radioligands: Impact of Minimal Structural Changes on the Tissue Distribution Profile

Luisa M. Deberle[1], Viviane J. Tschan[1], Francesca Borgna[1], Fan Sozzi-Guo[1], Peter Bernhardt[2,3], Roger Schibli[1,4], Cristina Müller[1,4*]

[1] *Center for Radiopharmaceutical Sciences ETH-PSI-USZ, Paul Scherrer Institute, 5232 Villigen-PSI, Switzerland*

[2] *Department of Radiation Physics, The Sahlgrenska Academy, University of Gothenburg, 413 45 Gothenburg, Sweden*

[3]*Department of Medical Physics and Medical Bioengineering, Sahlgrenska University Hospital, 413 45 Gothenburg, Sweden*

[4]*Department of Chemistry and Applied Biosciences, ETH Zurich, 8093 Zurich, Switzerland*

**E-Mail addresses**:
luisa.deberle@psi.ch; viviane.tschan@psi.ch; francesca.borgna@psi.ch; fan.sozzi@psi.ch; peter.bernhardt@gu.se; roger.schibli@psi.ch; cristina.mueller@psi.ch

***Correspondence to**:
PD Dr. Cristina Müller
Center for Radiopharmaceutical Sciences ETH-PSI-USZ
Paul Scherrer Institute
5232 Villigen-PSI
Switzerland
e-mail: cristina.mueller@psi.ch
phone: +41-56-310 44 54; fax: +41-56-310 28 49

## 1. HPLC Purification and Analysis of Ibu-PSMA-02

***Methods:*** High performance liquid chromatography (HPLC) of Ibu-PSMA-02 was performed for preparative and analytical purposes using a Merck-Hitachi LaChrom HPLC system, equipped with a D-7000 interface, a L-7200 autosampler, a L-7400 UV detector and a L-7100 pump.

The purification of the PSMA ligand was carried out using a semi-preparative reversed-phase C18 column (5 µm, 10×150 mm, Sunfire™, Waters, USA). A linear gradient of 0.1% (*v*/*v*) trifluoroacetic acid (TFA) in Milli-Q water (75–50%) and acetonitrile (MeCN) (25–50%) over 20 min was used at a flow rate of 4.0 mL/min. The fractions containing the product were collected and lyophilized to obtain the pure PSMA ligand as dried powder.

A reversed-phase C18 column (5 µm, 4.6 x 150 mm, Sunfire™, Waters, USA) was used for analytical purposes. In this case, a linear gradient of 0.1% (*v*/*v*) TFA in Milli-Q water (95–5%) and MeCN (5–95%) over 15 min was used at a flow rate of 1.0 mL/min.

***Results:*** Analytical data are presented in Table 1 of the main article.

## 2. Radiolabeling

***Methods:*** Ibu-PSMA-02 was dissolved in Milli-Q water and dimethylsulfoxide (25%) to obtain a final concentration of 1 mM. It was labeled with lutetium-177 (no-carrier-added, in 0.04 M HCl; ITM Medical Isotopes GmbH, Germany) in a 1:5 (*v/v*) mixture of sodium acetate (0.5 M, pH 8) and HCl (0.05 M, pH ~1) at pH ~4.5 [1,2]. Ibu-PSMA-02 was commonly prepared with a molar activity of 50 MBq/nmol if not otherwise stated in the article. The reaction mixture was incubated for 10 min at 95 °C, followed by quality control using HPLC. For this purpose, a Merck Hitachi LaChrom HPLC system, equipped with a D-7000 interface, a L-7200 autosampler, a radioactivity detector (LB 506 B; Berthold), a L-7100 pump and a reversed-phase C18 column (Xterra™ MS, C18, 5 µm, 150 x 4.6 mm; Waters) was used. The radiochemical purity was determined by calculating the peak areas of the radiolabeled product, potential unreacted lutetium-177 and radioactive species of unknown structure. A linear gradient of 0.1% (*v*/*v*) TFA in Milli-Q water (95–20%) and MeCN (5–80%) over 15 min was used at a flow rate of 1.0 mL/min. The reaction mixture was diluted in Milli-Q water containing sodium diethylenetriamine pentaacetic acid (Na-DTPA, 50 µM) prior to injection into HPLC.

***Results:*** The quality control resulted in high radiochemical purity (≥98%) of [$^{177}$Lu]Lu-Ibu-PSMA-02 labeled at a molar activity of up to 100 MBq/nmol. A representative HPLC chromatogram of [$^{177}$Lu]Lu-Ibu-PSMA-02 in comparison to previously published data of [$^{177}$Lu]Lu-Ibu-PSMA-01 and [$^{177}$Lu]Lu-PSMA-617 is shown in Figure S1.

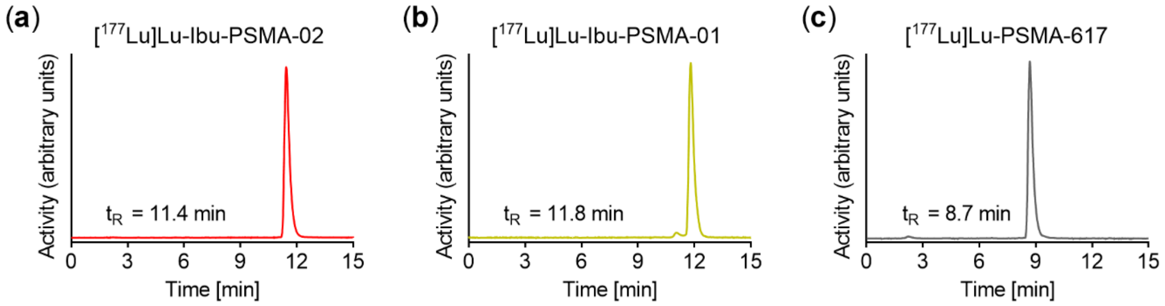


**Figure S1.** HPLC chromatograms of radioligands obtained as a result of the quality control. (**a**) [$^{177}$Lu]Lu-Ibu-PSMA-02; (**b**) [$^{177}$Lu]Lu-Ibu-PSMA-01 [3] and (**c**) [$^{177}$Lu]Lu-PSMA-617 [1,2]. The retention times ($t_R$) are indicated in the figures. The chromatogram of [$^{177}$Lu]Lu-PSMA-617 was adapted with permission from Benešová *et al.* 2018 Mol Pharm 15:934 [1]. Copyright 2020 American Chemical Society.

## 3. Radiolytic Stability

***Methods:*** Radiolytic stability of [$^{177}$Lu]Lu-Ibu-PSMA-02 was assessed over a period of 24 h. Ibu-PSMA-02 was labeled with lutetium-177 at a molar activity of 50 MBq/nmol. After quality control using HPLC (t = 0, radiochemical purity ≥ 98%, set to 100%), the labeling solution (~120 µL) was diluted with saline to an activity concentration of 250 MBq/500 µL with and without addition of L-ascorbic acid (3 mg). The radioligand solution was incubated at room temperature and the degradation was determined by investigation of an aliquot after 1 h, 4 h and 24 h incubation time using HPLC. The HPLC chromatograms were analyzed by integration of the peaks representing the radiolabeled product, the released lutetium-177 as well as degradation products of unknown structure. A quantitative assessment was performed by expressing the peak area of the intact product as percentage of the sum of integrated peak areas of the entire chromatogram.

***Results:*** Analysis of the radioligand solution after a 24 h incubation period without addition of L-ascorbic acid revealed >84% intact [$^{177}$Lu]Lu-Ibu-PSMA-02 indicating significantly increased stability as compared to the previously investigated [$^{177}$Lu]Lu-Ibu-PSMA-01 and [$^{177}$Lu]Lu-PSMA-617, respectively (Figure S2a). Complete stabilization of [$^{177}$Lu]Lu-Ibu-PSMA-02 was achieved by addition of L-ascorbic acid to the solution, which resulted in >96% intact radioligand after 24 h (Figure S2b).

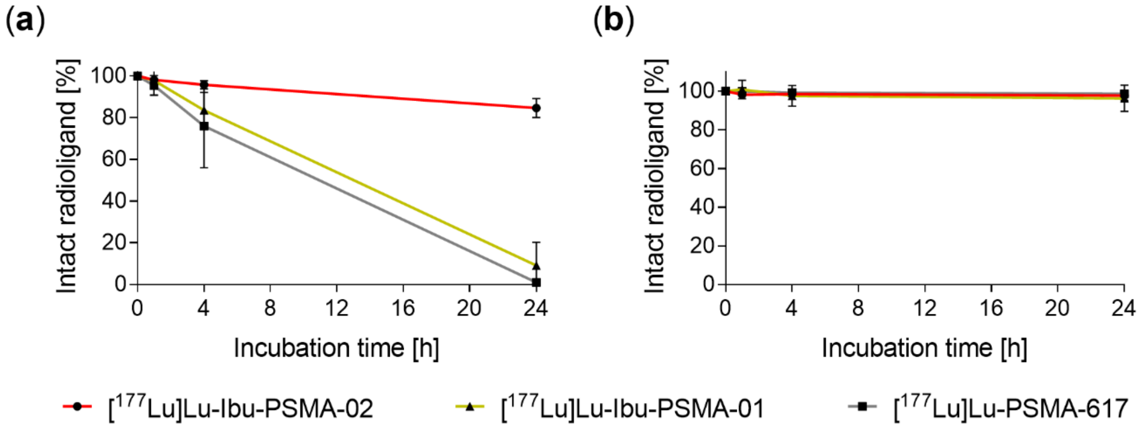


**Figure S2.** Radiolytic stability of [$^{177}$Lu]Lu-Ibu-PSMA-02, [$^{177}$Lu]Lu-Ibu-PSMA-01 [3] and [$^{177}$Lu]Lu-PSMA-617 [1]. (**a**) Radioligands incubated over a period of 24 h without L-ascorbic acid; (**b**) Radioligands incubated over a period of 24 h with L-ascorbic acid (average ± SD, n = 3). Data of [$^{177}$Lu]Lu-Ibu-PSMA-01 were reproduced from Deberle & Benešová *et al.* 2020 [3]. Data of [$^{177}$Lu]Lu-PSMA-617 were reproduced from Benešová *et al.* 2018 [1]. Copyright 2020 American Chemical Society.

## 4. Albumin-Binding Properties

***Methods:*** The investigation of the binding affinity to mouse plasma proteins is reported in the main article. The binding of the radioligands to human plasma proteins and pure human serum albumin (HSA) was investigated by adding the radioligand (15 µL, ~300 kBq, 0.006 nmol) to 150 µL human plasma (n = 1) or a HSA solution in PBS (700 µM, n = 3). The amount of radioligand bound to human plasma proteins or HSA was quantified using the same ultrafiltration method as reported in the main article for mouse plasma. The results are reported as percentage of radioligand bound to plasma proteins or HSA (Figure S3).

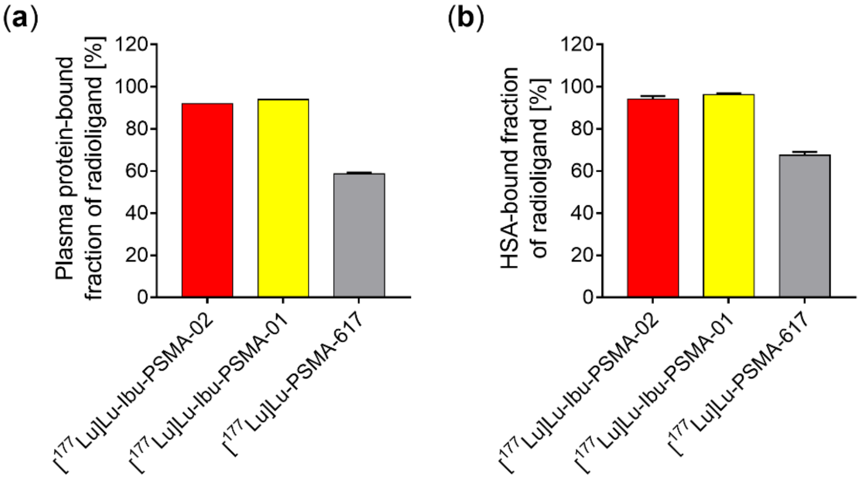


**Figure S3.** Plasma protein-binding properties of [$^{177}$Lu]Lu-Ibu-PSMA-02 in comparison to [$^{177}$Lu]Lu-Ibu-PSMA-01 and [$^{177}$Lu]Lu-PSMA-617. (**a**) Binding to human plasma proteins (n = 1); (**b**) Binding to pure HSA (n = 3). Binding data of [$^{177}$Lu]Lu-PSMA-617 to human plasma proteins were adapted with permission from Benešová *et al.* 2018 Mol Pharm 15:934 [2]. Copyright 2020 American Chemical Society.

***Results:*** It was revealed that the binding of [$^{177}$Lu]Lu-Ibu-PSMA-02 to human plasma proteins was the same as for [$^{177}$Lu]Lu-Ibu-PSMA-01 but stronger than for [$^{177}$Lu]Lu-PSMA-617. Comparable results were observed regarding the binding properties to pure HSA, indicating that the radioligands bound exclusively to serum albumin (Figure S3).

## 5. Biodistribution

***Methods:*** The methods of biodistribution studies are reported in the main article.

***Results:*** The results of the biodistribution studies are shown in Figure 4 of the main article and listed in Tables S1–S3. Tumor-to-background ratios of [$^{177}$Lu]Lu-Ibu-PSMA-02 and [$^{177}$Lu]Lu-Ibu-PSMA-01 are shown in Figure 5 of the main article. Tumor-to-kidney and tumor-to-liver ratios of all three radioligands are shown in Figure S4.

**Table S1.** Decay-corrected biodistribution data of [$^{177}$Lu]Lu-Ibu-PSMA-02 obtained in PC-3 PIP/flu tumor-bearing mice. The values represent the average value ± SD of the percentage injected activity per gram tissue [% IA/g] obtained from each group of mice (*n* = 4).

| | **[$^{177}$Lu]Lu-Ibu-PSMA-02** | | | | | |
|---|---|---|---|---|---|---|
| | **1 h p.i.** | **4 h p.i.** | **24 h p.i.** | **48 h p.i.** | **96 h p.i.** | **192 h p.i.** |
| Blood | 29 ± 4 | 7.1 ± 0.7 | 0.77 ± 0.08 | 0.35 ± 0.11 | 0.27 ± 0.01 | 0.03 ± 0.00 |
| Heart | 9.4 ± 1.5 | 2.4 ± 0.3 | 0.39 ± 0.04 | 0.18 ± 0.06 | 0.12 ± 0.01 | 0.02 ± 0.00 |
| Lung | 17 ± 3 | 4.8 ± 0.7 | 0.80 ± 0.07 | 0.37 ± 0.10 | 0.25 ± 0.03 | 0.05 ± 0.02 |
| Spleen | 5.8 ± 1.2 | 2.1 ± 0.3 | 0.72 ± 0.15 | 0.31 ± 0.08 | 0.22 ± 0.02 | 0.10 ± 0.01 |
| Kidneys | 115 ± 15 | 27 ± 2 | 10 ± 2 | 3.5 ± 0.7 | 2.3 ± 0.3 | 0.46 ± 0.04 |
| Stomach | 2.6 ± 0.5 | 1.3 ± 0.9 | 0.51 ± 0.19 | 0.23 ± 0.19 | 0.72 ± 0.81 | 0.03 ± 0.01 |
| Intestines | 3.0 ± 0.3 | 1.1 ± 0.2 | 0.32 ± 0.08 | 0.15 ± 0.06 | 0.13 ± 0.02 | 0.08 ± 0.06 |
| Liver | 17 ± 4 | 7.1 ± 0.5 | 0.82 ± 0.09 | 0.34 ± 0.12 | 0.21 ± 0.02 | 0.12 ± 0.03 |
| Salivary glands | 6.7 ± 0.5 | 1.9 ± 0.2 | 0.38 ± 0.05 | 0.15 ± 0.04 | 0.11 ± 0.01 | 0.02 ± 0.00 |
| Muscle | 3.2 ± 0.7 | 0.83 ± 0.14 | 0.14 ± 0.02 | 0.06 ± 0.01 | 0.04 ± 0.01 | 0.01 ± 0.00 |
| Bone | 3.3 ± 0.4 | 1.0 ± 0.2 | 0.27 ± 0.04 | 0.15 ± 0.04 | 0.08 ± 0.01 | 0.07 ± 0.01 |
| PC-3 PIP Tumor | 63 ± 8 | 98 ± 7 | 133 ± 15 | 73 ± 15 | 56 ± 9 | 24 ± 3 |
| PC-3 flu Tumor | 5.1 ± 0.7 | 2.0 ± 0.3 | 0.67 ± 0.07 | 0.27 ± 0.08 | 0.15 ± 0.01 | 0.06 ± 0.04 |
| Tumor-to-blood | 2.2 ± 0.1 | 14 ± 3 | 173 ± 18 | 216 ± 30 | 210 ± 35 | 918 ± 80 |
| Tumor-to-liver | 3.7 ± 0.5 | 14 ± 2 | 163 ± 16 | 224 ± 55 | 263 ± 42 | 209 ± 39 |
| Tumor-to-kidney | 0.6 ± 0.1 | 3.6 ± 0.2 | 13 ± 2 | 73 ± 15 | 25 ± 2 | 53 ± 7 |

**Table S2.** Decay-corrected biodistribution data of [$^{177}$Lu]Lu-Ibu-PSMA-01 obtained in PC-3 PIP/flu tumor-bearing mice. The values represent the average value ± SD of the percentage injected activity per gram tissue [% IA/g] obtained from each group of mice ($n$ = 3–6). Data were previously published by Deberle & Benešová *et al.* 2020 [3].

| | **[$^{177}$Lu]Lu-Ibu-PSMA-01** | | | | | |
|---|---|---|---|---|---|---|
| | **1 h p.i.** | **4 h p.i.** | **24 h p.i.** | **48 h p.i.** | **96 h p.i.** | **192 h p.i.** |
| Blood | 15 ± 2 | 6.0 ± 1.5 | 0.59 ± 0.11 | 0.50 ± 0.02 | 0.36 ± 0.06 | 0.15 ± 0.05 |
| Heart | 5.7 ± 1.3 | 2.3 ± 0.7 | 0.32 ± 0.06 | 0.26 ± 0.02 | 0.15 ± 0.02 | 0.08 ± 0.02 |
| Lung | 13 ± 6 | 3.7 ± 0.8 | 0.59 ± 0.13 | 0.51 ± 0.08 | 0.30 ± 0.03 | 0.15 ± 0.03 |
| Spleen | 3.4 ± 0.4 | 1.8 ± 0.3 | 0.67 ± 0.13 | 0.50 ± 0.04 | 0.35 ± 0.05 | 0.25 ± 0.03 |
| Kidneys | 30 ± 4 | 33 ± 1 | 16 ± 3 | 11 ± 1 | 4.1 ± 0.3 | 2.2 ± 0.5 |
| Stomach | 2.2 ± 0.9 | 0.79 ± 0.26 | 0.21 ± 0.03 | 0.15 ± 0.00 | 0.15 ± 0.05 | 0.10 ± 0.07 |
| Intestines | 2.4 ± 0.3 | 1.0 ± 0.2 | 0.21 ± 0.05 | 0.14 ± 0.01 | 0.09 ± 0.02 | 0.04 ± 0.01 |
| Liver | 5.9 ± 0.4 | 2.8 ± 0.5 | 0.95 ± 0.11 | 0.85 ± 0.06 | 0.67 ± 0.07 | 0.50 ± 0.01 |
| Salivary glands | 4.2 ± 0.4 | 1.7 ± 0.4 | 0.32 ± 0.07 | 0.26 ± 0.02 | 0.14 ± 0.02 | 0.08 ± 0.02 |
| Muscle | 2.1 ± 0.4 | 0.97 ± 0.37 | 0.11 ± 0.04 | 0.09 ± 0.02 | 0.05 ± 0.01 | 0.03 ± 0.01 |
| Bone | 2.2 ± 0.3 | 1.0 ± 0.2 | 0.17 ± 0.05 | 0.13 ± 0.01 | 0.09 ± 0.01 | 0.06 ± 0.01 |
| PC-3 PIP Tumor | 49 ± 6 | 81 ± 7 | 77 ± 21 | 58 ± 4 | 28 ± 2 | 17 ± 4 |
| PC-3 flu Tumor | 4.4 ± 1.3 | 2.2 ± 0.6 | 0.60 ± 0.19 | 0.51 ± 0.15 | 0.18 ± 0.01 | 0.08 ± 0.02 |
| Tumor-to-blood | 3.2 ± 0.6 | 14 ± 3 | 133 ± 29 | 116 ± 10 | 79 ± 9 | 116 ± 26 |
| Tumor-to-liver | 8.2 ± 0.4 | 30 ± 4 | 82 ± 21 | 68 ± 8 | 42 ± 4 | 33 ± 5 |
| Tumor-to-kidney | 1.6 ± 0.1 | 2.6 ± 0.1 | 4.9 ± 0.7 | 5.5 ± 0.4 | 6.8 ± 0.4 | 7.7 ± 0.2 |

**Table S3.** Decay-corrected biodistribution data of [$^{177}$Lu]Lu-PSMA-617 obtained in PC-3 PIP/flu tumor-bearing mice. The values represent the average value ± SD of the percentage injected activity per gram tissue [% IA/g] obtained from each group of mice ($n$ = 3–6). Data were reproduced from Benešová *et al.* 2018 [1]. Copyright 2020 American Chemical Society.

| | **[$^{177}$Lu]Lu-PSMA-617** | | | | | |
|---|---|---|---|---|---|---|
| | **1 h p.i.** | **4 h p.i.** | **24 h p.i.** | **48 h p.i.** | **96 h p.i.** | **192 h p.i.** |
| Blood | 0.50 ± 0.06 | 0.02 ± 0.33 | 0.01 ± 0.09 | 0.01 ± 0.00 | 0.01 ± 0.00 | 0.00 ± 0.00 |
| Heart | 0.29 ± 0.03 | 0.03 ± 0.22 | 0.01 ± 0.07 | 0.01 ± 0.00 | 0.00 ± 0.00 | 0.01 ± 0.00 |
| Lung | 0.57 ± 0.07 | 0.07 ± 0.08 | 0.03 ± 0.09 | 0.02 ± 0.00 | 0.01 ± 0.00 | 0.01 ± 0.00 |
| Spleen | 0.63 ± 0.27 | 0.15 ± 0.31 | 0.05 ± 0.11 | 0.04 ± 0.00 | 0.02 ± 0.01 | 0.03 ± 0.01 |
| Kidneys | 9.8 ± 1.4 | 3.7 ± 3.2 | 0.76 ± 0.92 | 0.35 ± 0.05 | 0.19 ± 0.04 | 0.16 ± 0.03 |
| Stomach | 0.18 ± 0.02 | 0.08 ± 0.09 | 0.03 ± 0.03 | 0.02 ± 0.00 | 0.01 ± 0.00 | 0.00 ± 0.00 |
| Intestines | 0.24 ± 0.00 | 0.07 ± 0.01 | 0.04 ± 0.04 | 0.01 ± 0.00 | 0.01 ± 0.00 | 0.00 ± 0.00 |
| Liver | 0.20 ± 0.04 | 0.09 ± 0.18 | 0.07 ± 0.09 | 0.04 ± 0.01 | 0.04 ± 0.01 | 0.04 ± 0.01 |
| Salivary glands | 0.42 ± 0.30 | 0.04 ± 0.01 | 0.02 ± 0.00 | 0.01 ± 0.01 | 0.03 ± 0.04 | 0.01 ± 0.00 |
| Muscle | 0.25 ± 0.20 | 0.02 ± 0.06 | 0.01 ± 0.04 | 0.01 ± 0.00 | 0.00 ± 0.00 | 0.00 ± 0.00 |
| Bone | 0.21 ± 0.04 | 0.06 ± 0.0.7 | 0.03 ± 0.02 | 0.03 ± 0.00 | 0.02 ± 0.00 | 0.02 ± 0.00 |
| PC-3 PIP Tumor | 44 ± 12 | 56 ± 8 | 37 ± 6 | 28 ± 4 | 21 ± 1 | 20 ± 4 |
| PC-3 flu Tumor | 0.28 ± 0.04 | 0.08 ± 0.01 | 0.05 ± 0.01 | 0.03 ± 0.00 | 0.02 ± 0.01 | 0.01 ± 0.00 |
| Tumor-to-blood | 88 ± 13 | 2315 ± 108 | 2730 ± 195 | 3776 ± 585 | 4019 ± 917 | 4316 ± 719 |
| Tumor-to-liver | 213 ± 17 | 598 ± 108 | 528 ± 51 | 710 ± 98 | 594 ± 153 | 528 ± 110 |
| Tumor-to-kidney | 4.5 ± 0.7 | 16 ± 3 | 50 ± 4 | 81 ± 11 | 112 ± 22 | 120 ± 3 |

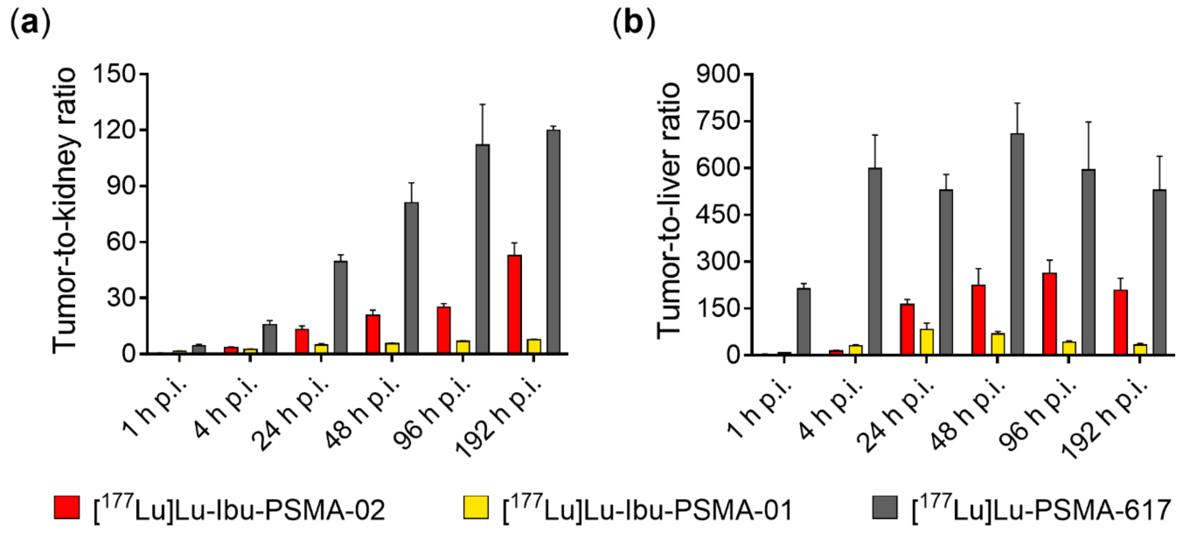


**Figure S4.** (**a**) Tumor-to-kidney ratios and (**b**) tumor-to-liver ratios at 1 h, 4 h, 24 h, 48 h, 96 h and 192 h after injection of [$^{177}$Lu]Lu-Ibu-PSMA-02 in comparison to [$^{177}$Lu]Lu-Ibu-PSMA-01 [3] and [$^{177}$Lu]Lu-PSMA-617 [1].

## 6. Area under the Curve (AUC) Values and AUC Ratios

***Methods:*** The area under the curve (AUC) was determined for the uptake of [$^{177}$Lu]Lu-Ibu-PSMA-02 in PC-3 PIP tumors and in the blood based on non-decay-corrected data obtained from the biodistribution data using GraphPad Prism software (version 7).

***Results:*** The AUC value representing the PC-3 PIP tumor uptake of [$^{177}$Lu]Lu-Ibu-PSMA-02 was almost 1.6-fold higher than the value of [$^{177}$Lu]Lu-Ibu-PSMA-01 (Figure S5).

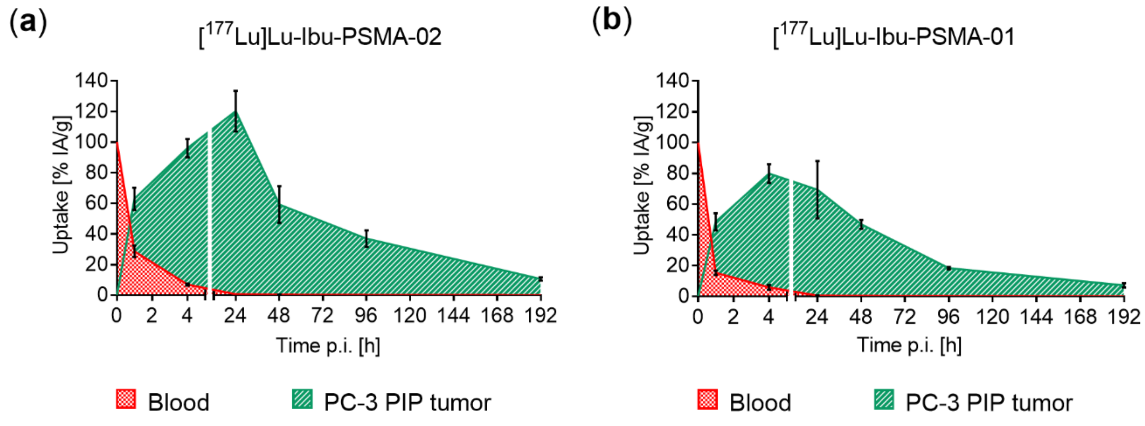


**Figure S5.** Area under the curves (AUCs) of [$^{177}$Lu]Lu-Ibu-PSMA-02 and [$^{177}$Lu]Lu-Ibu-PSMA-01. AUCs were calculated based on non-decay-corrected biodistribution data up to 192 h p.i. (8 d p.i.). (**a**) Tumor and blood AUCs of [$^{177}$Lu]Lu-Ibu-PSMA-02 and (**b**) Tumor and blood AUCs for [$^{177}$Lu]Lu-Ibu-PSMA-01 [3]. Each data point represents the average of a group of mice ± SD (n = 3−6) indicated as percentage of injected activity per gram tissue [% IA/g]. Data of [$^{177}$Lu]Lu-Ibu-PSMA-01 were reproduced from Deberle & Benešová *et al.* 2020 [3].

In comparison to [$^{177}$Lu]Lu-PSMA-617, the tumor AUC value of [$^{177}$Lu]Lu-Ibu-PSMA-02 was 2.5-fold increased, whereas the tumor AUC value of [$^{177}$Lu]Lu-Ibu-PSMA-01 was only 1.6-fold increased

(Table S4). On the other hand, [$^{177}$Lu]Lu-Ibu-PSMA-02 showed a 1.2-fold higher AUC value for the blood than [$^{177}$Lu]Lu-Ibu-PSMA-01 and both values were much higher than the blood AUC of [$^{177}$Lu]Lu-PSMA-617 (Table S4).

As a result of the increased tumor uptake of [$^{177}$Lu]Lu-Ibu-PSMA-02, the tumor-to-blood AUC ratio was 1.4-fold higher than the ratio obtained for [$^{177}$Lu]Lu-Ibu-PSMA-01, but 1.7-fold lower than for [$^{177}$Lu]Lu-PSMA-617 (Table S4).

**Table S4.** Area under the curve (AUC) values and ratios of AUCs based on non-decay-corrected biodistribution data.

| | [$^{177}$Lu]Lu-Ibu-PSMA-02 | [$^{177}$Lu]Lu-Ibu-PSMA-01[a] | [$^{177}$Lu]Lu-PSMA-617[b] |
|---|---|---|---|
| **AUC values [% IA/g·h]** | | | |
| PC-3 PIP tumor | 9172 ± 487 | 5896 ± 320 | 3691 ± 156 |
| Blood | 226 ± 10 | 194 ± 16 | 52 ± 2 |
| **Ratio of AUC values** | | | |
| $AUC_{Tu}$-to-$AUC_{Bl}$ | 41 ± 4 | 30 ± 5 | 71 ± 6 |

[a]Data of [$^{177}$Lu]Lu-Ibu-PSMA-01 were previously published by Deberle & Benešová *et al.* 2020 [3]. [b]Data of [$^{177}$Lu]Lu-PSMA-617 were reproduced from Benešová *et al.* 2018 [1]. Copyright 2020 American Chemical Society.

## 7. Whole-Body-Activity Measurements

***Methods:*** [$^{177}$Lu]Lu-Ibu-PSMA-02 (25 MBq/nmol) diluted in saline containing 0.05% BSA was intravenously injected into non-tumor bearing female, athymic nude BALB/c mice (25 MBq, 1 nmol, 100 µL; n = 3). The mice were measured immediately after injection and after emptying the urinary bladder at various timepoints over a period of 48 h p.i. The activity measured immediately after injection was set as 100%. The data points were based on non-decay-corrected results and presented as the average of the measured activity in three mice per radioligand ± standard deviation (SD). The results were compared with those previously obtained for [$^{177}$Lu]Lu-Ibu-PSMA-01 and [$^{177}$Lu]Lu-PSMA-617 [1,3].

***Results:*** The whole-body measurements revealed only slight differences in excretion patterns of [$^{177}$Lu]Lu-Ibu-PSMA-02 and [$^{177}$Lu]Lu-Ibu-PSMA-01 (Figure S6). Most of the injected [$^{177}$Lu]Lu-Ibu-PSMA-02 was still retained in the body 1 h after injection (89 ± 8%) and decreased to 55 ± 7% at 2 h p.i and 35 ± 5% at 4 h p.i. One day after injection, more than 95% of the initially injected activity was excreted. [$^{177}$Lu]Lu-Ibu-PSMA-01 was better retained in the body as indicated by higher values at all timepoints. On the other hand, [$^{177}$Lu]Lu-PSMA-617, which does not comprise a designated albumin-binding entity, was quickly excreted which resulted in <5% retained activity in the body at 2 h p.i.

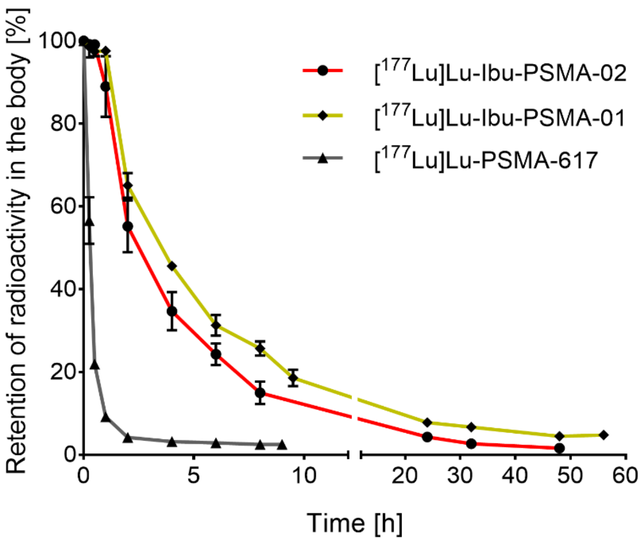


**Figure S6.** Whole-body activity measured at various timepoints after injection of the radioligands. The activity measured immediately after injection was set to 100%. Data of [$^{177}$Lu]Lu-Ibu-PSMA-01 were reproduced from Deberle & Benešová *et al.* 2020 [3]. Data of [$^{177}$Lu]Lu-PSMA-617 were reproduced from Benešová *et al.* 2018 [1]. Copyright 2020 American Chemical Society.

## 8. Synthesis of Ibu-PSMA-02

***Methods:*** Solvents and chemicals were purchased in analytical grade from commercial sources (Acros Organics, Alfa Aesar, Christof Senn Laboratories AG, Fisher Scientific, Fluorochem, Iris Biotech GmbH, Santa Cruz Biotechnology, Merck, Tokyo Chemical Industry) and used without further purification.

The glutamate-urea-based PSMA-binding entity – L-Glu-NH-CO-NH-L-Lys – was prepared on a 2-chlorotrityl chloride (2-CT) resin in analogy to the method described by Eder *et al.* [4]. The linker, which consisted of a 2-naphthyl-L-Ala and a *trans*-cyclohexyl moiety, was synthesized as previously reported by Benešová *et al.* to obtain compound **I** [5]. Resin-immobilized compound **I** was swelled in dry dichloromethane (DCM) for 45 min and conditioned in *N*,*N*-dimethylformamide (DMF). $N^{\alpha}$-Fmoc-$N^{\gamma}$-Alloc-L-2,4-diaminobutyric acid (Fmoc-DAB(Alloc)-OH, 0.4 mmol, 4.0 equiv) was activated with *O*-(benzotriazol-1-yl)-*N*,*N*,*N'*,*N'*-tetramethyluronium hexafluorophosphate (HBTU; 0.40 mmol, 3.96 equiv) in the presence of *N*,*N*-diisopropylethylamine (DIPEA; 0.80 mmol, 8.0 equiv) in anhydrous DMF and added to compound **I**. Compound **II** was obtained after agitation for 1 h. Removal of the Fmoc-protecting group by washing with 50% piperidine in DMF twice for 5 min yielded compound **III**. As previously reported by Umbricht *et al.* [2], the conjugation of the DOTA chelator to compound **III** was realized by adding a solution of 2-(4,7,10-tris(2-(t-butoxy)-2-oxoethyl)-1,4,7,10-tetraazacyclo-dodecan-1-yl)acetic acid (DOTA-(tris-*t*Bu) ester, 0.30 mmol, 3.0 equiv), HBTU (0.30 mmol, 2.97 equiv) and DIPEA (0.80 mmol, 8.0 equiv) in dry DMF to the resin and agitating for 5 h to obtain compound **IV**. The resin was then conditioned in DCM. For removal of the ε-Alloc-protecting group, tetrakis(triphenylphosphine)palladium(0) ($Pd(PPh_3)_4$, 0.03 mmol, 0.30 equiv) and morpholine (3 mmol,

30 equiv) were dissolved in dry DCM and added to compound **IV.** The reaction solution was agitated for 2 h in the dark to obtain precursor **1**. It was washed with DCM and, subsequently, with DMF. Precursor **1** was additionally washed with 1% DIPEA in DMF and then with a solution of sodium diethyldithiocarbamate (15 mg/mL) in DMF to remove residuals of palladium. The resin-immobilized precursor **1** was washed with DCM and diethylether and dried under reduced pressure.

**Scheme SI.** Solid-phase synthesis of precursor **1** used for the formation of Ibu-PSMA-02.

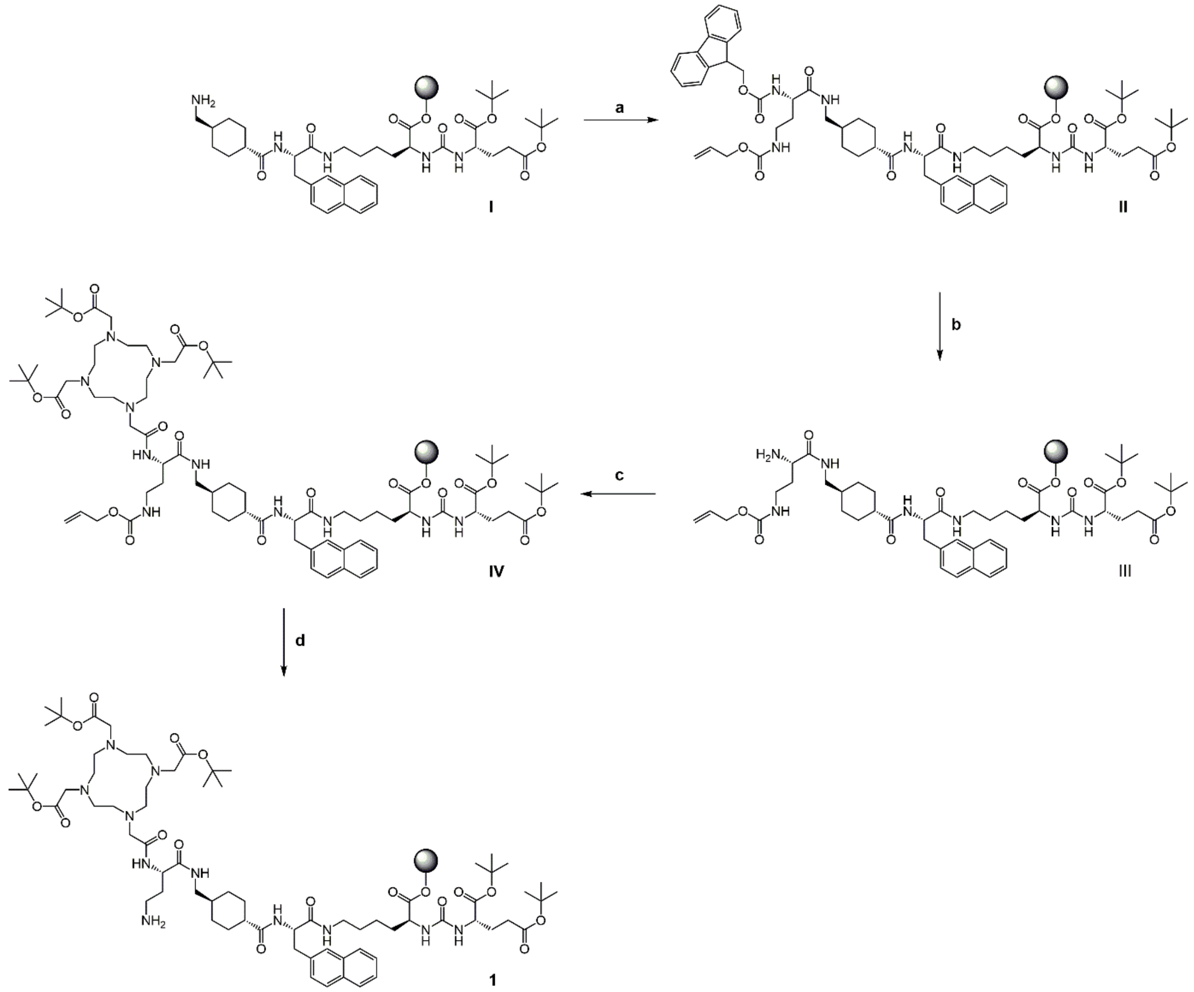

a) Fmoc-DAB(Alloc)-OH, HBTU, DIPEA, DMF; rt, 1 h; b) 50% Piperidine in DMF (v/v); rt, 2 x 5 min
c) DOTA-(tris-*t*Bu)ester, HBTU, DIPEA, DMF; rt, 5 h; d) $Pd(PPh_3)_4$, morpholine, DCM;rt, 2h

The resin-immobilized precursor **1** (0.10 mmol) was swelled in anhydrous DCM for 45 min and conditioned in DMF. A racemic mixture of 2-(4-(2-methylpropyl)phenyl)propanoic acid ((*RS*)-ibuprofen, 0.60 mmol, 6.0 equiv) was activated with HBTU (0.59 mmol, 5.94 equiv) in the presence of DIPEA (0.80 mmol, 8.0 equiv) in anhydrous DMF. The activated solution was added to precursor **1** and agitated for 1.5 h. The resulting resin-immobilized compound **2** was washed with DMF, DCM, and $Et_2O$ and dried under vacuum. The product was cleaved from the resin and simultaneously deprotected with a mixture consisting of TFA, triisopropylsilane (TIPS) and Milli-Q water in a ratio of 95:2.5:2.5 (*v/v*) within 3 h (main article, Scheme I). TFA was evaporated, the crude compound dissolved in MeCN and

Milli-Q water in a ratio of 1:1 (*v/v*) and purified by HPLC. The solution containing the pure product (Ibu-PSMA-02) was lyophilized followed by chemical characterization using analytical HPLC and MALDI-TOF-MS (Bruker UltraFlex II).

***Results:*** The results are reported in the main article (Table 1).

## 9. Cell Culture

Sublines of the androgen-independent PC-3 human prostate cancer cell line derived from an advanced androgen-independent bone metastasis had been created by transfection to express PSMA at high levels (PSMA-positive PC-3 PIP cells) or by mock transfection to obtain a cell line, which does not express PSMA (PSMA-negative PC-3 flu cells) [6]. Both cell lines have been used extensively by different groups, including our own, to evaluate a series of PSMA-targeting radiopharmaceuticals [1-3,7,8].
***Methods:*** Cells were grown in a humidified incubator at 37 ℃ and 5% $CO_2$ in RPMI-1640 cell culture medium supplemented with 10% fetal calf serum, L-glutamine, antibiotics, as well as puromycin (2 µg/mL) to maintain PSMA expression. Twice a week, cells were split using PBS/EDTA and trypsin for detachment in order to maintain the cell culture.

## 10. Dosimetric Calculations

***Methods:*** Bi-exponential functions were used to describe the tissue biokinetics of [$^{177}$Lu]Lu-Ibu-PSMA-02 and [$^{177}$Lu]Lu-Ibu-PSMA-01. Uncertainties in the time integrated activity concentration coefficients (TIACCs) were generated by using multiple unique biokinetic curves for tumor and kidney tissue, respectively. These curves were generated by selecting one of the measured tissue activity concentrations (MTACs) per time point only. Thereby, several unique biokinetic curves were generated by selecting different MTACs per timepoint for each curve. For each biokinetic curve a bi-exponential curve fit was performed using the software MATLAB (MathWorks, Torrance, California, USA). The TIACCs were obtained by integrating the generated bi-exponential functions to infinity. The mean and standard deviation of TIACC was calculated for [$^{177}$Lu]Lu-Ibu-PSMA-02 and [$^{177}$Lu]Lu-Ibu-PSMA-01. The specific mean absorbed dose $D$ for the PC-3 PIP tumor and kidneys was calculated by:

$$D = TIACC \cdot (\sum_i E_i \gamma_i \emptyset_i) \cdot \alpha \qquad (1)$$

where $E_i$ is the energy emitted of the $i^{th}$ radiation with a frequency per decay of $\gamma_i$; $\emptyset_i$ the absorbed energy fraction within an organ and $\alpha = 5.76 \cdot 10^{-7}$ the factor to convert to the unit Gy/MBq. The absorbed fractions were calculated by Monte Carlo simulation using PENELOPE 2014 [9]. In the simulations, spherical shapes of the organs were assumed. The decay data of lutetium-177 were obtained from ICRU 107 (www.nucleide.org).

***Results:*** The results are presented in the main article.

## References


1. Benešová, M.; Umbricht, C.A.; Schibli, R.; Müller, C. Albumin-binding PSMA ligands: optimization of the tissue distribution profile. *Mol Pharm* **2018**, *15*, 934-946, doi:10.1021/acs.molpharmaceut.7b00877.
2. Umbricht, C.A.; Benešová, M.; Schibli, R.; Müller, C. Preclinical development of novel PSMA-targeting radioligands: modulation of albumin-binding properties to improve prostate cancer therapy. *Mol Pharm* **2018**, *15*, 2297-2306, doi:10.1021/acs.molpharmaceut.8b00152.
3. Deberle, L.M.; Benešová, M.; Umbricht, C.A.; Borgna, F.; Büchler, M.; Zhernosekov, K.; Schibli, R.; Müller, C. Development of a new class of PSMA radioligands comprising ibuprofen as an albumin-binding entity. *Theranostics* **2020**, *10*, 1678-1693, doi:10.7150/thno.40482.
4. Eder, M.; Schäfer, M.; Bauder-Wüst, U.; Hull, W.E.; Wängler, C.; Mier, W.; Haberkorn, U.; Eisenhut, M. $^{68}$Ga-complex lipophilicity and the targeting property of a urea-based PSMA inhibitor for PET imaging. *Bioconjug Chem* **2012**, *23*, 688-697, doi:10.1021/bc200279b.
5. Benešová, M.; Schäfer, M.; Bauder-Wüst, U.; Afshar-Oromieh, A.; Kratochwil, C.; Mier, W.; Haberkorn, U.; Kopka, K.; Eder, M. Preclinical evaluation of a tailor-made DOTA-conjugated PSMA inhibitor with optimized linker moiety for imaging and endoradiotherapy of prostate cancer. *J Nucl Med* **2015**, *56*, 914-920, doi:10.2967/jnumed.114.147413.
6. Wu, P.; Kudrolli, T.A.; Chowdhury, W.H.; Liu, M.M.; Rodriguez, R.; Lupold, S.E. Adenovirus targeting to prostate-specific membrane antigen through virus-displayed, semirandom peptide library screening. *Cancer Res* **2010**, *70*, 9549-9553, doi:10.1158/0008-5472.CAN-10-1760.
7. Banerjee, S.R.; Pullambhatla, M.; Foss, C.A.; Nimmagadda, S.; Ferdani, R.; Anderson, C.J.; Mease, R.C.; Pomper, M.G. $^{64}$Cu-labeled inhibitors of prostate-specific membrane antigen for PET imaging of prostate cancer. *J Med Chem* **2014**, *57*, 2657-2669, doi:10.1021/jm401921j.
8. Choy, C.J.; Ling, X.; Geruntho, J.J.; Beyer, S.K.; Latoche, J.D.; Langton-Webster, B.; Anderson, C.J.; Berkman, C.E. $^{177}$Lu-Labeled phosphoramidate-based PSMA inhibitors: the effect of an albumin binder on biodistribution and therapeutic efficacy in prostate tumor-bearing mice. *Theranostics* **2017**, *7*, 1928-1939, doi:10.7150/thno.18719.
9. Salvat, F. PENELOPE2014: A code system for Monte-Carlo simulation of electron and photon transport. *OECD/NEA Data Bank: NEA/NSC/DOC* **2015**, *3*. https://www.oecd-nea.org/science/docs/2015/nsc-doc2015-3.pdf
